# Synergistic reduction in interfacial flexibility of TREM2^R47H^ and ApoE4 may underlie AD pathology

**DOI:** 10.1002/alz.70120

**Published:** 2025-04-12

**Authors:** Emma E. Lietzke, David Saeb, Emma C. Aldrich, Kimberley D. Bruce, Kayla G. Sprenger

**Affiliations:** ^1^ Department of Chemical and Biological Engineering University of Colorado Boulder Boulder Colorado USA; ^2^ Division of Endocrinology Metabolism, and Diabetes University of Colorado Anschutz Medical Campus Aurora Colorado USA

**Keywords:** Alzheimer's disease, ApoE, interfacial flexibility, molecular dynamics, TREM2

## Abstract

**BACKGROUND:**

The strongest genetic drivers of late‐onset Alzheimer's disease (AD) are apolipoprotein E4 (ApoE4) and TREM2^R47H^. Despite their critical roles, the mechanisms underlying their interactions remain poorly understood.

**METHODS:**

We conducted microsecond‐long molecular dynamics simulations of TREM2‐ApoE complexes, including TREM2^R47H^, validating our findings through comparison with published experimental data on TREM2‐ApoE binding interactions.

**RESULTS:**

Our simulations reveal TREM2^WT^ can sample an “open” CDR2 conformation, challenging the prevailing notion that this conformation is pathogenic. TREM2^WT^ exhibits greater flexibility, accessing diverse CDR2 conformations, while rigidity in TREM2^R47H^’s CDR2 may explain its reduced ligand‐binding properties. ApoE2 facilitates dynamic reconfiguration of TREM2‐ApoE2 complexes, which is absent with ApoE4. TREM2^R47H^ and ApoE4 mutually rigidify each other, suppressing interfacial flexibility.

**DISCUSSION:**

Our findings suggest mechanisms underlying ApoE2's neuroprotective functions, ApoE4's pathogenicity, and the synergistic effects of ApoE4 and TREM2^R47H^ in AD. TREM2^WT^’s flexibility and reconfiguration with ApoE2 may support microglial activation, while rigidity in TREM2^R47H^‐ApoE4 interactions may drive pathogenic signaling.

**Highlights:**

- TREM2^WT^ samples diverse CDR2 conformations, challenging prior assumptions that an “open” CDR2 state is solely pathogenic.
- ApoE2 promotes dynamic reconfiguration of TREM2‐ApoE2 complexes, preserving TREM2^WT^'s flexibility.
- ApoE4's hinge forms a unique binding pocket that enhances TREM2 binding.
- The TREM2^R47H^‐ApoE4 complex exhibits mutual rigidity, suppressing CDR2 and hinge flexibility.

## 1 BACKGROUND

Alzheimer's disease (AD) is a progressive neurodegenerative disorder affecting nearly seven million Americans.^1^ Many factors contributing to AD risk have been identified,^2^, ^3^, ^4^, ^5^, ^6^ and microglia, the brain's resident macrophages, play a crucial role in AD pathology.^7^, ^8^ Microglia phagocytose amyloid‐β aggregates, process myelin‐derived lipids and lipoproteins, and release cytokines that can either protect or damage neurons and glia.^9^ Key proteins involved in these processes include apolipoprotein E (ApoE), a lipid transport protein, and triggering receptor expressed on myeloid cells 2 (TREM2), a microglial transmembrane glycoprotein that binds diverse ligands—including ApoE^10,11^—mediating microglial activation, signaling, and effector functions such as phagocytosis.^12^, ^13^ Despite the established roles of ApoE and TREM2 in microglial function and AD pathology, their precise residue‐level interactions remain unclear.

TREM2 features an immunoglobulin (Ig)‐like extracellular domain with three complementarity‐determining regions (CDRs), which provide multiple ligand‐binding surfaces.^14^ The AD‐risk variant TREM2^R47H^—one of the strongest risk factors for late‐onset AD, second only to the ε4 allele of ApoE (ApoE4)^15^, ^16^—is thought to impair ligand binding by altering TREM2's CDR2 structure,^17^ compromising microglial function.^14^, ^17^, ^18^, ^19^ However, the exact molecular mechanisms remain undefined. Similarly, TREM2's broader role in neurodegenerative diseases remains complex. Studies suggest increased TREM2 expression on disease‐associated microglia may rescue dysfunctional lipid processing,^20^, ^21^ while chronic TREM2 overactivation could elevate brain inflammation.^22^ TREM2's diverse ligand‐binding capabilities, including its interactions with ApoE, may underlie these seemingly conflicting roles, especially as ApoE levels and lipidation state change during disease progression.^23^, ^24^ ApoE4, in particular, may synergistically exacerbate AD pathology through maladaptive interactions with TREM2^R47H^, though the underlying mechanisms remain elusive.^25^

ApoE facilitates lipid transport by acting as a major scaffold protein in peripheral and brain‐derived lipoproteins.^26^, ^27^ Its three canonical isoforms differ by a single‐residue polymorphism from the most common isoform, ApoE3 (C112/R158). ApoE2 (C112/C158) exhibits protective functions against AD, while ApoE4 (R112/R158) is a major driver of late‐onset AD.^15^ A recent study found *APOE4* homozygotes had higher levels of AD biomarkers and increased AD pathology compared to *APOE3* homozygotes.^28^ Structural studies indicate ApoE's N‐terminal domain mediates receptor binding, while its C‐terminal domain facilitates lipid binding and self‐association. These domains are connected by a hinge region, which may facilitate lipid binding by regulating their relative movement.^29^ The single‐residue change in unlipidated ApoE4 is thought to induce large structural changes, hindering its proper function.^6^, ^30^, ^31^ However, this remains uncertain, as only an unlipidated, monomeric nuclear magnetic resonance (NMR) structure of ApoE3 has been determined, with five artificial mutations introduced to prevent aggregation^32^.

Currently, there are no resolved, published structures of unlipidated ApoE2 and ApoE4, any lipidated ApoE isoform, or ApoE's conformation within lipoproteins. However, transmission electron microscopy studies revealed that astrocyte‐secreted, lipidated ApoE adopts an antiparallel orientation on discoidal lipoproteins, regardless of ApoE isoform.^33^ Furthermore, cryogenic electron microscopy confirmed that two lipidated ApoE4 molecules form an antiparallel “double‐belt” conformation in lipoproteins.^33^ While this study indicates lipidation‐mediated ApoE structural changes are similar to other apolipoproteins, it did not yield structures suitable for future biophysical studies.

**RESEARCH IN CONTEXT**

1. **Systematic review**: We conducted a thorough literature review using PubMed and relevant conference proceedings. Most prior studies on TREM2 and apolipoprotein E (ApoE) in Alzheimer's disease (AD) focus on experimental models, with limited computational work that primarily includes TREM2‐only models or short timescales. The molecular dynamics of TREM2‐ApoE interactions and the synergistic effects of pathogenic variants like TREM2^R47H^ and ApoE4 remain largely unexplored.
2. **Interpretation**: Our findings reveal how TREM2^R47H^ and ApoE4 interactions exacerbate AD pathology. Stabilized secondary structures in both proteins reduce interfacial flexibility, suppressing necessary conformational changes and limiting ligand engagement. This rigidity may explain a synergistic mechanism contributing to AD progression.
3. **Future directions**: Future research should investigate how ApoE lipidation impacts TREM2 binding and whether alternative ligands can counteract pathogenic rigidity. Beyond biophysical assays, membrane‐bound TREM2 models are essential for validating these computational insights and enhancing physiological relevance.

Consequently, substantial gaps remain in understanding how ApoE binds to receptors and how its conformation and lipidation status affect binding. Although ApoE has known receptor and lipid‐binding sites, a recent biophysical study suggested an uncharacterized ApoE hinge region mediates binding with TREM2.^34^ Two molecular dynamics (MD) studies of ApoE‐TREM2 interactions have also been conducted.^35^, ^36^ One broadly recapitulated trends of increased ApoE4‐TREM2 binding and decreased ApoE3‐TREM2^R47H^ binding^35^ but did not observe expected structural changes due to short simulation times and retaining the artificial mutations in the resolved ApoE3 structure.^35^ The other study showed that ApoE3‐TREM2^WT^ binding affinity decreases in the presence of lipids,^36^ but the proteins were not equilibrated before docking and simulations were again too brief to capture anticipated structural changes. To elucidate these detailed structural changes with critical implications for immune regulation, we conducted the longest MD simulations of TREM2 and TREM2‐ApoE complexes to date, offering unprecedented insights into their interaction dynamics to advance AD understanding and therapeutic design.

## 2 METHODS

### 2.1 Structural model preparation

The crystal structures of human wild‐type (WT) TREM2 (TREM2^WT^) and TREM2^R47H^, as well as the NMR structure of human ApoE3, were retrieved from the Protein Data Bank (PDB) with the codes 5ELI, 5UD8, and 2L7B, respectively.^18^, ^19^, ^32^ The TREM2^WT^ and TREM2^R47H^ crystal structures comprise the extracellular, Ig‐like domain of TREM2 (residues N20‐D131 and H19‐A130, respectively). However, the TREM2^R47H^ crystal structure is unmodeled from R76‐S81; therefore, the homology‐modeling software MODELLER was utilized to insert these residues into the crystal structure.^37^, ^38^, ^39^, ^40^. The NMR structure of ApoE3 includes residues K1‐H299 (numbered 19‐317) and contains five point mutations in the C‐terminal region (F257A, W264R, V269A, L279Q, and V287E; numbered using 1‐299).^32^ These mutations were reverted to the WT residues using PyMOL 2.5,^41^ which was also employed to introduce the appropriate polymorphisms into the respective ApoE isoforms (ApoE2: C112/C158; ApoE3: C112/R158; ApoE4: R112/R158).

### 2.2 Pre‐docking MD simulations

All simulations were performed using the GROMACS 2023 software.^42^ The CHARMM36 force field^43^, ^44^, ^45^, ^46^, ^47^, ^48^ was employed to model proteins and ions, while the TIP3P‐modified force field was used for water.^49^ Energy minimization was initially conducted in vacuum and again after the addition of water and neutralizing ions to relax the systems. Subsequently, MD equilibration in the NVT ensemble was carried out for 1 ns using a modified Berendsen thermostat^50^ at 310 K. This was followed by an NPT equilibration step, also for 1 ns, conducted using the same thermostat and the Berendsen barostat^50^ at 310 K and 1 bar. Production simulations were then independently performed for each system (TREM2^WT^, TREM2^R47H^, ApoE2, ApoE3, and ApoE4) in triplicate for 1 µs each, resulting in a total production simulation sampling time of 15 µs prior to docking. All production simulations were carried out with the Berendsen thermostat^50^ and the Parrinello‐Rahman barostat^51^, ^52^, ^53^ at 310 K and 1 bar, employing a 2‐fs timestep and full periodic boundary conditions, with a neighbor list cutoff of 1.4 nm. Bonds involving hydrogen atoms were constrained using the LINCS algorithm.^54^ The particle mesh Ewald (PME) summation method was used to calculate long‐range electrostatic interactions beyond a short‐range cutoff value of 1.2 nm.^55^ A van der Waals cutoff value of 1.2 nm was also applied.

### 2.3 Selection of structures for docking

The root‐mean‐square deviation (RMSD) of the Cα atoms in each protein was measured over time, in reference to their respective energy‐minimized structures, to assess for structural stability and convergence during the production MD simulations. Additionally, the root‐mean‐square fluctuation (RMSF) of the Cα atoms in each protein was measured and averaged across the entire simulation period. Similarly, the reference structure for these calculations was the respective protein after energy minimization.

For TREM2, two distances were defined and calculated to assess conformational changes in the CDR2. “Loop 1” refers to the distance between the Cα atoms of R46 and L71, used to evaluate how far the CDR2 moved away from the rest of the Ig‐like domain. “Loop 2” represents the distance between the Cα atoms of H67 and W78, used to assess shape changes within the CDR2 throughout the simulation. Loop 2 was plotted against Loop 1, and linear regression fits were performed to identify any correlations between these distances. To explore the variable conformations of TREM2 dependent on CDR2, clustering was performed based on Loop 1 distance using GROMACS clustering tools.^40^, ^56^ The mean Loop 1 distance and its standard deviation were calculated across the entire simulation period for each TREM2 variant's replicates. These values were used to cluster TREM2 conformations into three groups: Cluster A (distances more than one standard deviation above the mean), Cluster B (distances within the mean ± one standard deviation), and Cluster C (distances more than one standard deviation below the mean). Representative structures from each cluster were then identified for both TREM2^WT^ (WT‐A, WT‐B, WT‐C) and TREM2^R47H^ (R47H‐A, R47H‐B, R47H‐C), and subsequently selected for docking.

For ApoE, the same clustering algorithm^40^, ^56^ was employed to identify the most representative structure of each ApoE isoform across their respective replicates. These structures were then used for docking analyses. Secondary structures of ApoE were also analyzed using Ramachandran plots. The GROMACS rama algorithm^40^, ^57^ was utilized to determine the phi and psi angles of Cα atoms over the converged portions of the simulations (every 0.5 ns for the last 100 ns). These angles were plotted and mapped according to the constraints associated with various secondary structure motifs, such as β‐sheets, right‐handed (RH) α‐helices, and left‐handed (LH) α‐helices. The number of residues in each ApoE replicate (per isoform) involved in these secondary structure motifs was counted and plotted over time, alongside the true average for each ApoE isoform. Analysis of variance (ANOVA) and pairwise Tukey honest significance difference (HSD) tests were conducted on the overall true mean (averaged over the last 100 ns) to determine significant differences in residues involved in the various secondary structure motifs across ApoE isoforms, particularly in the canonical and extended hinge regions and the C‐terminal region.

### 2.4 Protein‐protein docking

The online docking server ClusPro 2.0 was utilized for rigid protein‐protein docking.^58^, ^59^, ^60^, ^61^ Eighteen separate docking simulations were conducted for the following structures: WT‐A, WT‐B, WT‐C, R47H‐A, R47H‐B, and R47H‐C, each paired with representative, average structures of ApoE2, ApoE3, and ApoE4. ClusPro 2.0 generated 20 to 30 models for each protein‐protein pair. The top five scoring models were assessed for biological relevance using visual molecular dynamics (VMD),^62^ determined by analyzing TREM2 CDR residues (CDR1: aa40‐47, CDR2: aa67‐78, CDR3: aa115‐120) within 4.0 Å of ApoE hinge residues (aa167‐231), as defined by Kober et al.^34^ These identified residues were compared to those reported in the literature via in vitro binding assays^34^ as being involved in binding. Thus, these residues were used as a filter, such that the top model with the highest percentage of total TREM2 CDR residues within 4.0 Å of ApoE hinge residues was selected for post‐docking MD simulations. Models were visually inspected in VMD, and those with artifacts (spuriously interconnected loops) were excluded from selection.

### 2.5 Post‐docking MD simulations

For each TREM2‐ApoE pair, one model from ClusPro 2.0 underwent a 500‐ns MD simulation, adding 9 µs of production simulation time (total of 21 µs). The same workflow and parameters described earlier for pre‐docking MD simulations were applied to the docked complexes.

### 2.6 Simulation trajectory analysis

Model assessment entailed analyzing structural changes in TREM2 throughout the simulations. Standard GROMACS tools were employed to calculate the RMSD and RMSF of Cα atoms in both ApoE and TREM2. As described above, similar analyses of the Loop 1 and Loop 2 distances in TREM2 were performed over the course of the simulations.

The interactions between ApoE and TREM2 were assessed by estimating the interaction free energies of the complexes using the molecular mechanics Poisson‐Boltzmann surface area (MM/PBSA) method. This analysis employed the *gmx_MMPBSA* tool implemented in GROMACS,^63^, ^64^ which was performed over the last 50 ns of each simulation (450–500 ns). To assess the energies, a free energy was calculated for each TREM2‐ApoE complex every 0.5 ns for each simulation (*n* = 1818 data points). To further classify the interaction energies, each data point was paired with its corresponding TREM2 variant, ApoE isoform, and Loop 1 distance of TREM2 at that specific time point. Based on the value of Loop 1, energies were classified into the previously defined Clusters A, B, or C, for both TREM2^WT^ and TREM2^R47H^. Energies were plotted as boxplots with standard deviations, and two‐way ANOVA with Tukey HSD multiple comparison tests were performed for all groups. The distances between residues for both ApoE and TREM2 over the last 50 ns of each simulation were calculated using the MDAnalysis Python library.^65^, ^66^ Distances were plotted in contact maps for the respective TREM2 and ApoE models. These residues were compared to those identified from binding assays in existing literature.^34^ This comparative analysis across all TREM2‐ApoE models aimed to elucidate differences in binding impacted by TREM2 conformation, the R47H mutation, and ApoE isoform.

## 3 RESULTS

### 3.1 Broad suppression of CDR2 dynamics, versus simply an open CDR2 conformation, may impair TREM2^R47H^'s ability to bind and respond to diverse ligands

In our recent past work, we characterized binding between soluble TREM2 (sTREM2), TREM2, and phospholipids, noting a seemingly stochastic transition of TREM2^WT^’s CDR2 from a closed to an open conformation over the course of a 1 µs simulation.^67^ Previous experimental and MD studies have suggested that the mutation‐induced opening of CDR2 may disrupt structural integrity and impair ligand binding, as is observed in the presence of disease‐risk TREM2 variants Y38C, R47H, W50S, W50C, R52C, R62H, T66M, N68K, D86 V, T96K, D104G, and V126G.^14^, ^18^, ^19^, ^68^, ^69^ However, none of these previous studies simulated these structures or those of TREM2^WT^ for longer than 500 ns, and therefore had not observed similarly increased flexibility in the WT. We hypothesized that TREM2^WT^ could frequently access open CDR2 configurations in 1 µs simulations, as we did in our prior TREM2 and sTREM2 study,^67^ and that this flexibility would create variable binding sites for ApoE binding and challenge the notion that an open CDR2 is always detrimental to ligand binding.

**FIGURE 1:**
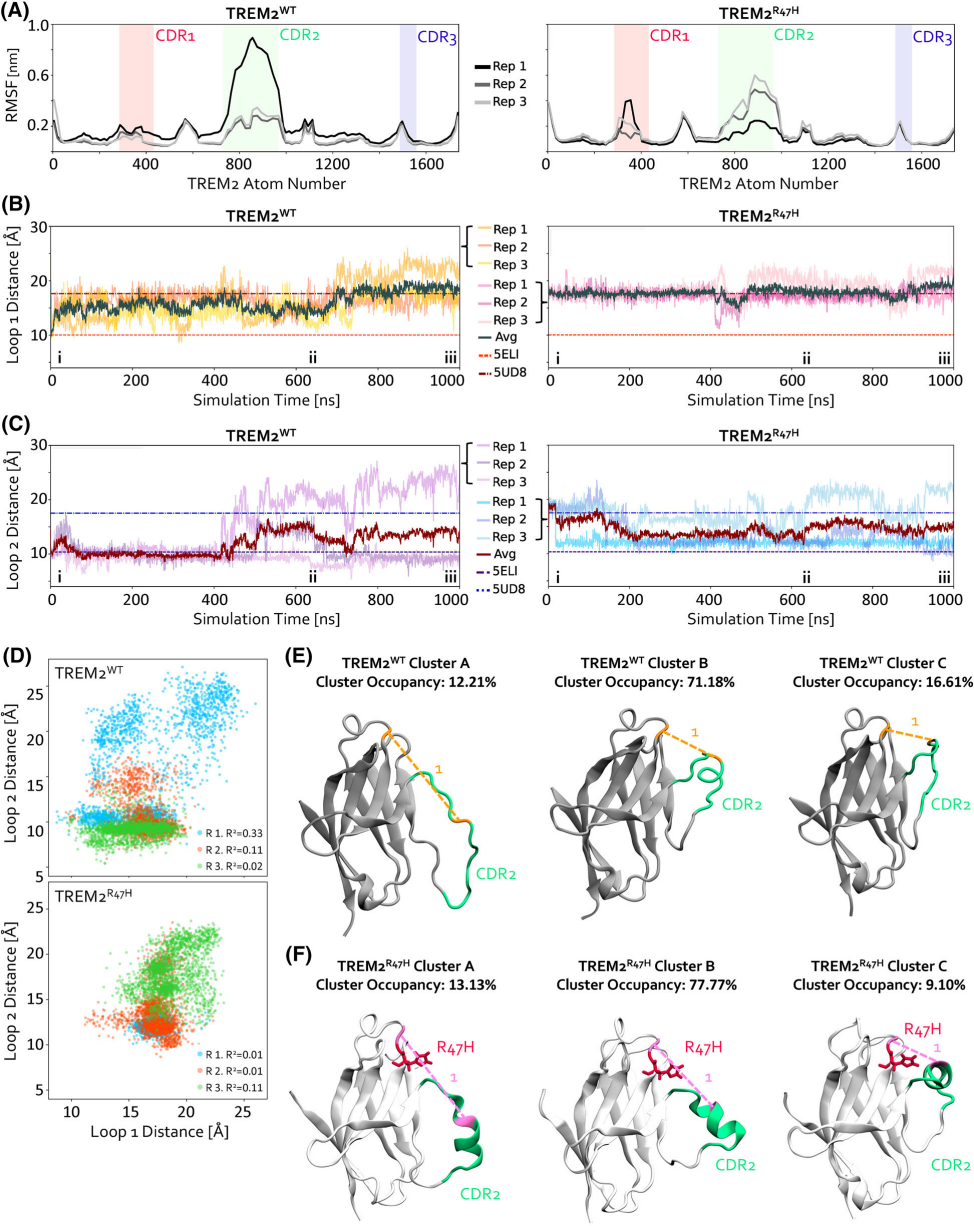
TREM2 samples diverse CDR2 conformations over long timescale simulations. (A) Cα RMSF vs. atom number for TREM2^WT^ and TREM2^R47H^ simulations. CDRs are shown in red, green, and blue. (B,C) Loop 1 [R46‐L71; (B)] and Loop 2 [H67‐W78; (C)] distances vs. time for TREM2^WT^ (left) and TREM2^R47H^ (right) simulations. Static loop distances measured from the crystal structures of TREM2^WT^ (PDB 5ELI) and TREM2^R47H^ (PDB 5UD8) are included as dotted lines. (D) Scatterplots of temporally paired Loop 2 vs. Loop 1 distances for all TREM2^WT^ (top) and TREM2^R47H^ (bottom) simulations. The R‐squared value for each individual replicate is shown, after linear regression fitting. (E‐F) Representative images of trajectory snapshots of the TREM2 structure for Clusters A, B, and C for TREM2^WT^ (top) and TREM2^R47H^ (bottom). TREM2^WT^ is shown in light gray, with CDR2 in green, and the Loop 1 residues in orange. TREM2^R47H^ is shown in white, with CDR2 in green, the Loop 1 residues in pink, and the R47H mutation in red, in licorice representation.

First, TREM2^WT^ and TREM2^R47H^ were simulated in an aqueous solvent for 1 µs in triplicate (see Figure S1A for RMSD profiles). RMSF calculations were performed over the course of the simulations. One of the three TREM2^WT^ replicates exhibited substantially higher RMSF values in the CDR2 region than the other replicates (Figure 1A, left). Interestingly, the maximum RMSF values observed with TREM2^WT^ surpassed those recorded for any of the three TREM2^R47H^ replicates (Figure 1A, right). These observations support the notion that large structural and dynamical changes in the CDR2 occur stochastically in the TREM2^WT^ model, but not in TREM2^R47H^ (Figure 1A).

To characterize the temporal changes in CDR2 behavior between TREM2^WT^ and TREM2^R47H^, we quantified distances between CDR2 and TREM2's remaining Ig‐like domain, which contains other relevant ligand binding residues. Visual analysis of our simulation trajectories in VMD revealed two potentially relevant CDR2‐based metrics, designated as Loop 1 (R46‐L71) and Loop 2 (H67‐W78), which we measured for all three replicates of TREM2^WT^ (Figure 1B‐C, left) and TREM2^R47H^ (Figure 1B‐C, right). The Loop 1 distance metric assesses how far CDR2 extends from the rest of TREM2's Ig‐like domain. Loop 1 distance remained stable across all three TREM2^R47H^ simulations (Figure 1B, right), exhibiting an average distance consistent with that observed in the TREM2^R47H^ crystal structure (PDB 5UD8). Conversely, the Loop 1 distance exhibited substantially greater dynamic behavior in all TREM2^WT^ simulations, even surpassing the static Loop 1 distance observed in the TREM2^R47H^ crystal structure by the end of the simulations (Figure 1B, left). As such, we postulated that the increased flexibility of CDR2 may not directly reduce ligand binding, but that a less dynamic CDR2, seen in TREM2^R47H^, may impair TREM2's ability to respond to diverse ligands.

The Loop 2 distance metric assesses how CDR2's conformation and dynamics change during the simulations. Both TREM2^WT^ (Figure 1C, left) and TREM2^R47H^ (Figure 1C, right) accessed previously unreported CDR2 conformations, described by Loop 2 distances exceeding 20 Å. Furthermore, a direct correlation between changes in Loop 1 and Loop 2 distances was not observed (Figure 1D), suggesting that the flexibility of CDR2, both in its ability to extend from the rest of TREM2's Ig‐like domain and its relative regional openness, can be independently characterized.

Further molecular‐level insights into our results were obtained through analysis of trajectory snapshots of TREM2^WT^ (Figure 1D; see Figure S1C for Loop labeling) and TREM2^R47H^ (Figure 1E; see Figure S1D for Loop labeling). TREM2^R47H^ maintained α‐helicity in CDR2 across all replicates [Figure S1D (i‐iii)], consistent with prior literature.^14^, ^19^ In contrast, TREM2^WT^’s CDR2 exhibited variable α‐helicity, with one simulation entirely losing α‐helicity, resulting in large fluctuations in CDR2 movement [Figure S1C (i‐iii)]. In the other two TREM2^WT^ simulations, the CDR2 maintained less α‐helicity than TREM2^R47H^. Furthermore, these patterns are relatively consistent with previous work that identified specific interacting residues between CDR1 and CDR2 in TREM2^WT^ and TREM2^R47H^ structures.^19^ In TREM2^WT^, we analyzed residues experimentally shown to participate in H‐bonding networks (R47, K48, S65, T66, H67, N68), while in TREM2^R47H^, we examined residues known to engage in π–π stacking (H47, H67) and H‐bonding (H47, T66).^19^ Interestingly, we found that interactions between residue 47 and additional residues in the backbone of CDR2 (aa65‐68) coincided with the presence of an α‐helix in CDR2, although not all tracked residues were consistently part of this network. In the sole simulation of TREM2^WT^ that lost its α‐helix in CDR2—resulting in increased CDR2 flexibility—we observed a corresponding loss of H‐bonding at this critical junction between CDR1 and CDR2 (Figure S1E).

We then analyzed residue‐level interactions at this same juncture in TREM2^R47H^. The orientation of H47 and H67 consistently suggested π–π stacking, while H‐bonding between H47 and T66 was preserved. Notably, we observed additional H‐bonding involving K48 and S65, which has not been previously reported in the literature.^19^ Taken together, these observations suggest that interactions at the junction between CDR1 and CDR2 contribute to maintaining CDR2 rigidity and that the *R47H* mutation enhances this structural property, which may fluctuate stochastically in TREM2^WT^. Moreover, while TREM2^R47H^’s CDR2 can access an open conformation, maintenance of the α‐helix may restrict CDR2 flexibility. This increased rigidity in TREM2^R47H^’s CDR2 could explain its reduced binding affinities for ligands, like ApoE, compared to TREM2^WT^, as observed in vitro.^17^, ^34^ These findings suggest that suppression of CDR2 dynamics in TREM2^R47H^, rather than simply a more open CDR2 conformation as the previous literature would suggest,^14^, ^19^, ^68^, ^69^ may reduce its ligand binding affinities and prevent binding to diverse ligands, leading to pathogenic TREM2‐mediated signaling and functions.

### 3.2 Loss of secondary structure in the ApoE canonical hinge region across all isoforms suggests an α‐helix in the extended hinge region primarily mediates TREM2 binding

Since TREM2^WT^’s CDR2 is more flexible than TREM2^R47H^’s CDR2, we hypothesized that this flexibility impacts ApoE binding. To test this, we employed long‐timescale simulations with unlipidated ApoE2, ApoE3, and ApoE4 to characterize conformational changes between ApoE isoforms before investigating TREM2‐ApoE interactions. As we simulated TREM2 for 1 µs in triplicate, we wanted to ensure ApoE isoforms were simulated under identical conditions prior to studying their interactions. In addition, of the two previous MD studies of these interactions, one used ApoE isoforms that contained the five point mutations in the C‐terminal of the ApoE3 crystal structure and only simulated ApoE in solvent for 20 ns,^35^ while the other did not simulate ApoE3 prior to docking.^36^ Therefore, to simulate the physiologically relevant form of unlipidated ApoE for longer timescales, we reverted these mutations in ApoE2, ApoE3, and ApoE4, and simulated each of these isoforms for 1 µs each in triplicate.

**FIGURE 2:**
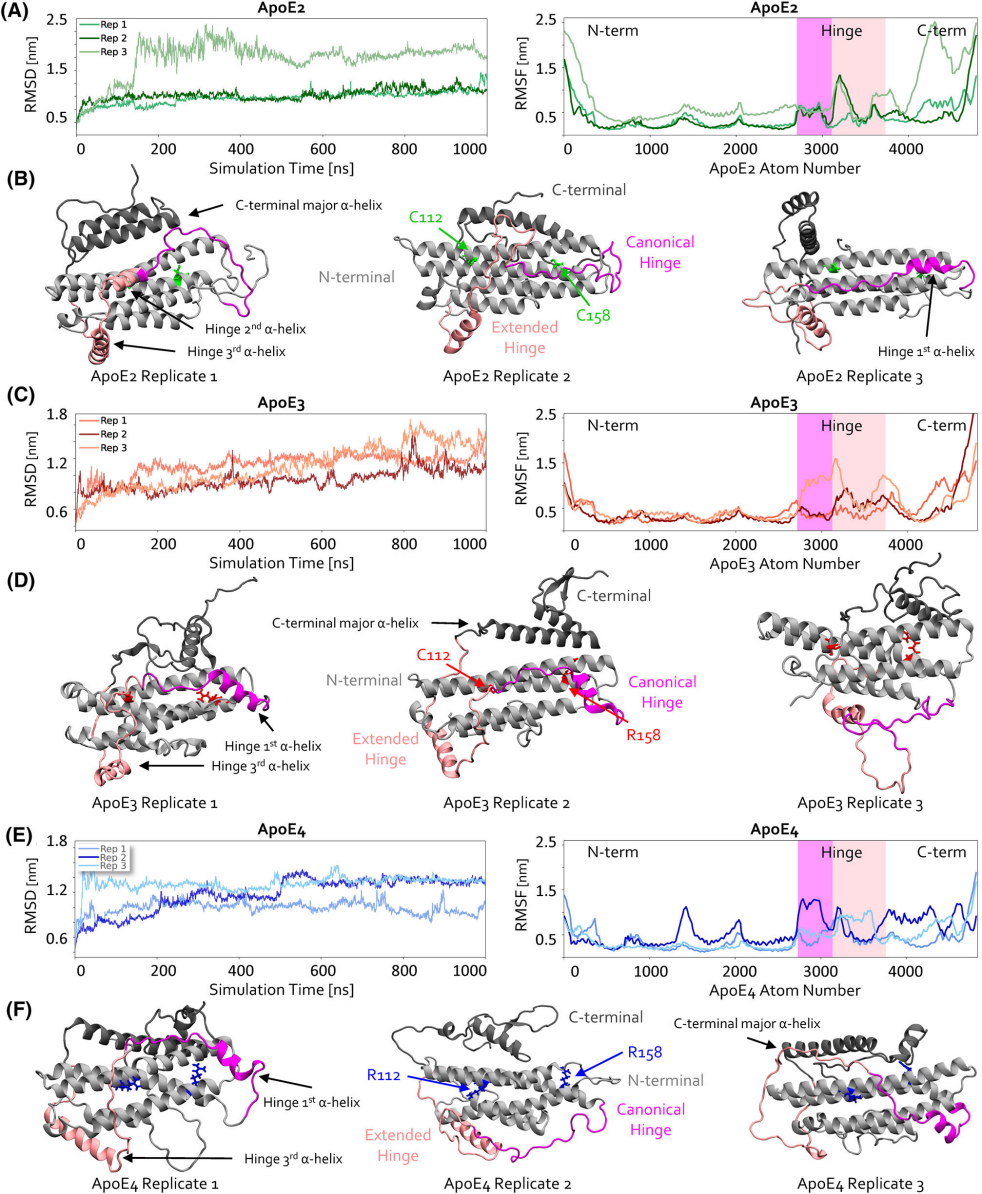
The stability of ApoE's hinge and C‐terminal regions varies greatly across isoforms. (A, C, E) RMSD (left) and RMSF (right) vs. time and atom number, resp., for ApoE2 (A), ApoE3 (C), and ApoE4 (E) simulations. (B, D, F) Structures of ApoE2 (B), ApoE3 (D), and ApoE4 (F), shown at their final simulation timepoints (1000 ns). Regions of ApoE are highlighted, with the canonical hinge (aa167‐191) colored in magenta, the extended hinge (aa192‐231) in light pink, the N‐terminal in light gray, and the C‐terminal in dark gray. Isoform‐differentiating residues are shown in green for ApoE2 (C112/C158), red for ApoE3 (C112/R158), and blue for ApoE4 (R112/R158).

Simulations were assessed for convergence by measuring RMSD (see the Methods section), which was achieved for all simulations by 900 ns (Figure 2A,C,E, left). The 900‐ to 1000‐ns period was used to assess conformational changes in stable protein forms. RMSF was measured across the entire simulation time to measure the flexible regions that induced changes in stability (Figure 2A,C,E, right). Visual analysis of ApoE isoforms showed a distinct loss of α‐helical content, particularly in the entire canonical hinge region (aa167‐191) and into the extended hinge region within the C‐terminal. Kober et al. described this extension of the hinge into the C‐terminal, prior to the lipid binding site, which we label as the extended hinge region (aa192‐231), relevant to TREM2 binding.^34^ The ApoE3 crystal structure comprises three α‐helices in this region: one in the canonical hinge (E168‐L181), another at the junction of the canonical and extended hinges (V190‐G200), and the third in the extended hinge (A209‐R224). Reduced α‐helical content was observed primarily in the two helices in the junctional and canonical hinge regions. These results align with experimental findings showing flexible and intrinsically disordered regions near the ApoE hinge,^29^ which are thought to sense environmental cues and mediate the protein's internal dynamics—mechanisms essential for lipid binding and adopting varied conformational states. In contrast, the α‐helix in the extended hinge maintained structural integrity across all simulations. This trend was consistent across all ApoE isoforms, further implicating that the extended hinge region may be important for TREM2 binding due to its more stable, helical binding interface.

Visual analysis of our simulation trajectories in VMD also revealed small β‐sheets emerging within disordered C‐terminal regions in several simulations, as shown in the final trajectory snapshots in Figure 2. When this occurred, β‐sheet structures manifested in the region proximal to the C‐terminal α‐helix. To verify these observations and more broadly assess structural changes in the hinge and C‐terminal regions, we tracked the number of residues in a β‐sheet, right‐handed (RH) α‐helix, and left‐handed (LH) α‐helix over the last 100 ns of each ApoE replicate simulation (see Figure S2B for hinge and Figure S3B for C‐terminal analysis).

Notably, the hinge region of ApoE4 was observed to contain the most residues likely to participate in β‐sheet formation and the fewest residues likely to be involved in RH α‐helix formation, suggesting a possible structural or energetic trade‐off between these two secondary structure motifs (Figure S2A, *p* < 0.001). In the remaining C‐terminal region, ApoE3 was found to have significantly more residues likely to participate in β‐sheet formation and fewer residues likely to be involved in RH α‐helix formation compared to ApoE2 and ApoE4 (Figure S3A, *p* < 0.001). Additionally, ApoE3 contained the highest number of residues likely to participate in LH α‐helix formation in this region. These trends indicate that even slight differences in the propensity for forming various secondary structure motifs in the hinge and C‐terminal regions could influence ApoE's ability to bind to different receptors, such as TREM2, and affect its self‐association behavior. Furthermore, variations in secondary structure across the three ApoE isoforms are reflected in the average representative structures of each ApoE isoform, obtained via clustering (Figure S4A; see the Methods section for details), which we believe are the most physiologically relevant structures of unlipidated ApoE to date.

### 3.3 The relatively weak binding affinity of ApoE2 for TREM2^WT^ facilitates dynamic reconfiguration of TREM2^WT^‐ApoE2 complexes, unlike with ApoE4

**FIGURE 3:**
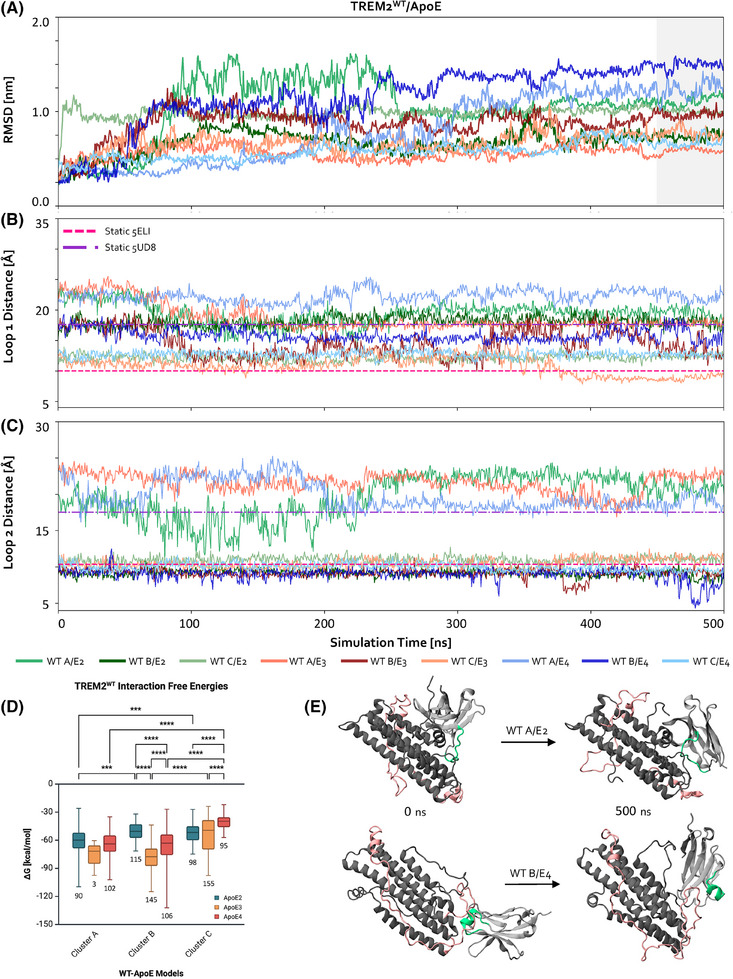
TREM2^WT^ can maintain distinct CDR2 conformations over time when in contact with ApoE. (A) RMSD vs. time for the nine simulations (WT‐A/B/C with ApoE2/3/4). The last 50 ns of the simulation, which was used for interaction energy and contact map calculations, is colored in gray. (B) Loop 1 distances of TREM2^WT^ models vs. time. The distances of references structures 5ELI (TREM2^WT^) and 5UD8 (TREM2^R47H^) are in hot pink and purple dashed lines, respectively. (C) Loop 2 distances of TREM2^WT^ models vs. time. (D) Interaction free energies of TREM2^WT^‐ApoE models from 450‐500 ns of the nine simulations (n = 909 simulation frames). True means with standard deviations and ranges are plotted in TREM2^WT^ Clusters A/B/C per ApoE isoforms. The number of frames per group are listed under each box plot. Two‐way ANOVA with Tukey HSD multiple comparison tests were performed for all groups, with some of the selected significant differences (*** *p* < 0.001, **** *p* < 0.0001) are shown. E) Trajectory snapshots at 0 and 500 ns of WT‐A/ApoE2 (top) and WT‐B/ApoE4 (bottom). Proteins are pictured in new cartoon representation. TREM2^WT^ is shown in light gray, with CDR2 in green. ApoE is shown in dark gray, with the canonical and extended hinge regions in light pink.

In our previous results, we observed novel CDR2‐based TREM2 conformations and generated biologically relevant forms of ApoE. Here, we aimed to determine how these new structures would interact, and more specifically, how the observed variations in structure in TREM2's CDR2 and across ApoE isoforms impact this receptor‐ligand interaction. To do so, we performed clustering analyses on both the TREM2 and ApoE simulation trajectories. This generated three TREM2 conformations (Figure 1D; Figure 1E), where “A” represents a conformation with the most open and largest Loop 1 distance, “B” denotes an intermediate distance, and “C” signifies the most closed and smallest distance (Figure S1B). Subsequently, each of the three selected TREM2^WT^ structures (WT‐A, WT‐B, WT‐C; see the Methods section) was docked using ClusPro 2.0 to the representative, temporally‐averaged structures of ApoE2, ApoE3, and ApoE4 (Figure S4A). For ensuing MD simulations, we selected the most biologically relevant complex from the top five scoring outputs in each case (Figure S5). This resulted in a total of nine simulations, each performed for 500 ns. To assess convergence, the RMSD of the full complex was tracked over time (Figure 3A).

Initial docked positions and TREM2‐ApoE contacts were analyzed using VMD to determine if there was a TREM2^WT^ conformation that consistently bound ApoE differently. From the initial docking positions, we observed that WT‐A bound to a different site on ApoE2, ApoE3, and ApoE4, compared to WT‐B and WT‐C. TREM2^WT^ generally remained bound to these same initial binding sites across ApoE isoforms, though its CDRs occasionally reconfigured their positioning at the binding site. This finding might suggest that TREM2^WT^ has a broadly open state (A) that impacts its ability to bind to the same ligands at different sites.

To test this energetically, we determined the interaction free energies between each TREM2‐ApoE pair over the last 50 ns of the simulations (Figure 3D; see Figure S6A for all statistics). We found no significant differences in the WT‐A interaction energies across ApoE isoforms, which is intriguing given that, as mentioned above, WT‐A binds to a unique site on each isoform. Notably, ApoE3 sampled the WT‐A conformation only rarely, suggesting that WT‐A is unlikely to be the preferred binding conformation for this isoform. In contrast, we observed significant differences in WT‐B interactions across all ApoE isoforms, with ApoE2 displaying the weakest interactions with both WT‐A and WT‐B. Lastly, the binding affinities between WT‐C and ApoE4 were significantly lower than those with ApoE2 or ApoE3. This suggests WT‐C's conformation is particularly suboptimal for ApoE4 and that ApoE4 preferentially binds to more open TREM2^WT^ CDR2 conformations, which are the most frequently accessed states of TREM2^WT^ (Figure 1E). Indeed, the strongest binding interactions observed in the simulations occurred between WT‐B and ApoE4.

We then questioned if the behavior of Loop 1 and Loop 2 was driving the differential binding between WT‐A and ApoE. Thus, we tracked the Loop 1 and Loop 2 distances across each TREM2^WT^ simulation (Figure 3B and 3C, respectively). In most cases, the distances remained fairly constant throughout the simulation, suggesting that once TREM2^WT^ binds to ApoE, the structure of CDR2 stabilizes and does not fluctuate between open and closed states, indicating reduced flexibility of TREM2^WT^ and, thus, an overall more stable interaction complex. Upon determining that TREM2^WT^’s CDR2 structure stabilizes during ApoE binding interactions, we examined whether ApoE's structure also stabilized when bound to TREM2^WT^. We found that compared to ApoE3 and ApoE4, ApoE2 exhibited a more flexible C‐terminal, with the end of this region (∼aa285‐299) lacking secondary structure and moving away from the remaining structure (Figure S7A, top row). Conversely, ApoE4's initial representative structure contained a more clustered hinge region, in which its extended hinge had more self‐interactions and created a pocket that TREM2^WT^ could bind to; this pocket was maintained throughout the course of the TREM2^WT^‐ApoE4 simulations, regardless of TREM2^WT^’s conformation.

Finally, another interesting trend emerged when analyzing the RMSF of ApoE across the simulations. The extended hinge region in all ApoE4 simulations showed greater flexibility than those of ApoE2 and ApoE3 (Figure S7A, top row). This is significant, considering that the extended hinge region was identified as the primary TREM2 binding site. Furthermore, the increased flexibility observed for ApoE4 could be a product of ApoE4's self‐interactions within this isoform‐unique binding pocket or increased TREM2^WT^ interactions at this site, although these behaviors could also depend on one another and thus have a synergistic effect on ApoE4 hinge behavior.

To better understand the observed stabilization of TREM2^WT^ and ApoE upon binding, we analyzed the simulation trajectories to assess how the overall complex's dynamics evolved throughout the simulations. In two of the three ApoE2 simulations, both TREM2^WT^ and ApoE2 underwent notable dynamic reconfigurations, with TREM2^WT^ shifting away from its initial binding site to engage a different region of ApoE2. This shift involved new interaction sites on TREM2^WT^ that were not previously in contact with ApoE2. In contrast, ApoE3 simulations demonstrated stable binding, with TREM2^WT^ largely maintaining its initial binding site and consistently involving the same CDRs as in the original configuration. Meanwhile, in the ApoE4 simulations, the proteins exhibited some degree of movement, though this was localized to the ApoE4 hinge pocket, the primary TREM2^WT^‐ApoE binding interface. Interestingly, these localized conformational shifts in both proteins appeared to increase contact between TREM2^WT^’s CDRs and ApoE4's hinge region, while preserving the overall binding site, suggesting a dynamic yet stabilizing interaction.

Collectively, these results suggest that ApoE isoforms have specific conformations that are ideal or unideal for binding to TREM2^WT^ and that ApoE2 can more readily dynamically shift to achieve one of these desired states. This also suggests that the conformational changes in ApoE required for lipid binding may still occur, particularly for ApoE2 interactions with TREM2^WT^, potentially explaining ApoE2's neuroprotective functions.^70^, ^71^ In contrast, ApoE4 appears to resist the dynamic reconfiguration observed with ApoE2. This resistance may be attributed to our observation of ApoE4's highly structured TREM2‐binding pocket in its extended hinge, which, as supported by prior experiments,^32^ facilitates the strongest interactions between the two proteins.

Finally, to assess how these variations in not only the secondary structure of ApoE but also its ability to dynamically shift on a tertiary level impact interactions with TREM2^WT^, contact maps were determined to see if the binding between TREM2^WT^ and ApoE was occurring at the different interfaces (e.g., hinge regions, lipid and receptor binding region; Figure S8). Despite changes in ApoE conformation or isoform, TREM2^WT^ consistently interacted with ApoE at its hydrophobic tip and CDRs. However, the regions on ApoE that interacted with TREM2^WT^ varied greatly. Generally, TREM2^WT^ interactions with ApoE2 involved its canonical hinge region and N‐terminal. With ApoE3, interactions involved its C‐terminal, whereas with ApoE4, interactions involved the extended hinge region and N‐terminal. These interaction differences across ApoE isoforms may suggest a mechanism underlying the stronger TREM2^WT^‐ApoE4 interactions observed in vitro^31^: even in the unlipidated, monomeric form, TREM2^WT^ appears to preferentially bind the extended hinge region pocket of ApoE4, a feature not observed with ApoE2 or ApoE3.

### 3.4 TREM2^R47H^ and ApoE4 when bound exacerbate rigidity at their CDRs and hinge regions, reflecting a limited ability for dynamic reconfiguration that may contribute to AD pathology

Experimental work has shown varying results regarding the binding of ApoE and TREM2 variants, depending on the method, TREM2 protein source, and ApoE lipidation state. Most studies indicate that TREM2 and ApoE are high‐affinity binders, with the majority reporting differences in binding across ApoE isoforms,^10^, ^11^, ^34^, ^72^, ^73^ while one study observed no significant differences in sTREM2‐Fc‐ApoE binding.^74^ Several studies using ELISA, dot blot, and BLI have shown that TREM2^R47H^ binding is greatly reduced across ApoE isoforms,^10^, ^11^, ^72^ whereas others, also employing BLI, reported only minor decreases.^34^, ^73^ To explore whether our previously observed differences in CDR2 behavior might explain this behavior, we performed experiments identical to the ones described in the previous section, except now between our three TREM2^R47H^ models with varying Loop 1 distances (R47H‐A, R47H‐B, R47H‐C; see the Methods section) and representative ApoE isoform models, resulting in nine total simulations between the three conformations of TREM2^R47H^ (Figure 1F) and representative ApoE isoforms (Figure S4).

**FIGURE 4:**
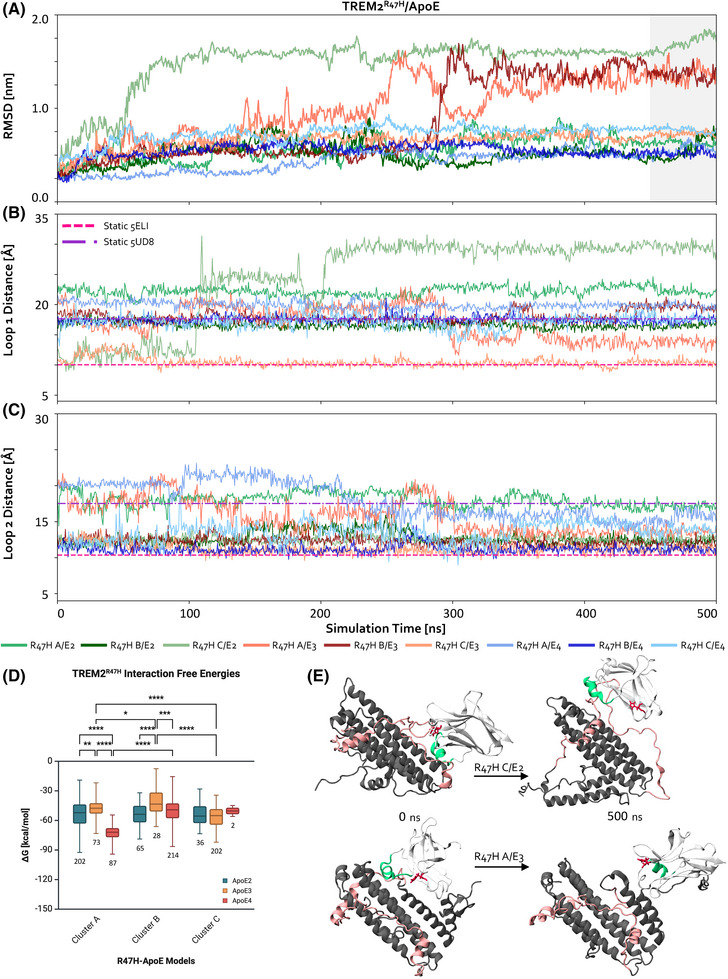
TREM2^R47H^ converges at similar CDR2 conformations over time despite initial varying states. (A) RMSD vs. time for the nine simulations (R47H‐A/B/C with ApoE2/3/4). The last 50 ns of the simulation, which was used for interaction energy and contact map calculations, is colored in gray. (B) Loop 1 distances of TREM2^R47H^ models vs. time. The distances of references structures 5ELI (TREM2^WT^) and 5UD8 (TREM2^R47H^) are in hot pink and purple dashed lines, respectively. (C) Loop 2 distances of TREM2^R47H^ models vs. time. (D) Interaction free energies of TREM2^R47H^‐ApoE models from 450‐500 ns of the nine simulations (*n* = 909 simulation frames). True means with standard deviations and ranges are plotted in TREM2^R47H^ Clusters A/B/C per ApoE isoforms. The number of frames per group are listed under each box plot. Two‐way ANOVA with Tukey HSD multiple comparison tests were performed for all groups, with some of the selected significant differences (* *p* < 0.05, ** *p* < 0.01, *** *p* < 0.001, **** *p* < 0.0001) are shown. (E) Trajectory snapshots at 0 and 500 ns of R47H‐C/ApoE2 (top) and R47H‐A/ApoE3 (bottom). Proteins are pictured in new cartoon representation. TREM2^R47H^ is shown in light gray, with CDR2 in green and the R47H mutation in red, with licorice representation. ApoE is shown in dark gray, with the canonical and extended hinge regions in light pink.

The RMSD of each complex was plotted to assess convergence (Figure 4A). Since we previously observed that the most open Loop 1 conformation of TREM2^WT^ (WT‐A) bound to distinct sites across ApoE isoforms (despite similar interaction energies), we investigated whether TREM2^R47H^ exhibited similar behavior. To test this, we compared the final MD‐equilibrated binding sites of the three TREM2^R47H^ models for each ApoE isoform, along with their corresponding interaction free energies (Figure 4D; see Figure S6B for all statistics). A distinct trend emerged: specific TREM2^R47H^ Loop 1 conformations were sampled more frequently when bound to certain ApoE isoforms. ApoE2 sampled the R47H‐A conformation most often, ApoE3 the R47H‐C conformation, and ApoE4 the R47H‐B conformation. In contrast, the TREM2^WT^ simulations showed a more even sampling of the three Loop 1 conformations across all ApoE isoforms. These findings suggest that TREM2^R47H^ adopts unique, isoform‐specific conformations, whereas TREM2^WT^ exhibits greater adaptability to ApoE. The *R47H* mutation restricts this adaptability, allowing only specific combinations of the conformations of the two proteins to be readily accessed. By comparison, TREM2^WT^’s pre‐binding adaptability allows it to form diverse binding interfaces across the different ApoE isoforms.

Given TREM2^R47H^’s reduced adaptability to ApoE, we questioned whether any single TREM2^R47H^ conformation might exhibit greater capacity for adaptation than others, as indicated by similar binding affinities and no strong preference for a given isoform. Indeed, there were no significant differences in the interaction energies of R47H‐C across all ApoE isoforms, directly contrasting with the behavior observed for WT‐C. Furthermore, the interaction energies of R47H‐A differed significantly across ApoE isoforms, indicating a reduced capacity to broadly accommodate ApoE, which is also the opposite of WT‐A, as reiterated above.

Notably, the frequency of sampling different TREM^R47H^ conformations does not consistently align with trends in interaction energies. For ApoE3, the most frequently sampled conformation corresponds to the strongest interaction energy, whereas for ApoE4, it corresponds to one of the weaker interactions. For ApoE2, no significant differences in interaction energy are observed across the different TREM^R47H^ conformations. These trends suggest that ApoE2 may rely more on conformational flexibility and adaptability, while ApoE3 and ApoE4 interact through more rigid or energetically defined pathways. This is consistent with previous results for TREM2^WT^, where ApoE2's weaker initial binding allowed dynamic reconfiguration of the TREM2^WT^‐ApoE2 complex to achieve ideal conformations, whereas ApoE3 and ApoE4 were less able to dynamically reconfigure. Collectively, these results suggest that while ApoE exhibits consistent isoform‐specific interaction mechanisms, TREM2^WT^’s enhanced adaptability enables stable complexes across all isoforms, while TREM2^R47H^’s restricted adaptability amplifies the isoform‐specific mechanisms of ApoE.

While the above differences in Loop 1 conformations and interaction energies provide insights into TREM2^R47H^’s isoform‐specific binding, we questioned if changes in Loop 1 and 2 distances over time could further explain the mechanisms underlying its experimentally observed reduced binding affinity for ApoE. To investigate this, we tracked these distances during the simulations (Figure 4B,C), following the method used for TREM2^WT^. Like TREM2^WT^, TREM2^R47H^ exhibited variable Loop 1 distances. In contrast, the Loop 2 distance across all TREM2^R47H^ simulations consistently ended at values below that of the static reference structure (PDB 5UD8). Furthermore, this convergence of Loop 2 distance was unique to TREM2^R47H^. We hypothesized that Loop 2 distances might depend on the amount of α‐helicity observed in CDR2. Since TREM2^WT^ not only has less α‐helicity but also sometimes loses this secondary structure altogether, this hypothesis appears consistent, as the WT‐A Loop 2 distances remained relatively close to their initial values.

Since we previously observed shifts in ApoE2's structure upon interaction with TREM2^WT^, we investigated whether similar structural changes occurred with TREM2^R47H^. Two simulations revealed large structural changes in ApoE2 and ApoE3, where the C‐terminal began to unfold and move away from the clustered helices, occasionally resulting in increased interactions with TREM2^R47H^. These conformational changes are further supported by increased RMSF values for ApoE2 and ApoE3, indicating significant flexibility in CTD residues compared to simulations with TREM2^WT^ (Figure S7A, bottom row). While this behavior is thought to occur when ApoE becomes lipidated,^29^, ^75^ it remains unclear why receptor interactions would induce similar changes. Notably, this behavior was only observed with ApoE2 (TREM2^WT^ and TREM2^R47H^) and ApoE3 (TREM2^R47H^), but never with ApoE4. Furthermore, we observed decreases in the RMSF of extended hinge region residues in ApoE4 (Figure S7A, bottom row). Importantly, this region displayed elevated RMSF values exclusively for ApoE4 bound to TREM2^WT^. In contrast, these residues exhibited reduced flexibility in the presence of TREM2^R47H^. The reduced flexibility in the extended hinge region, seemingly induced by TREM2^R47H^, may explain the significantly decreased binding affinity of ApoE4 for TREM2^R47H^ compared to TREM2^WT^.

To further investigate this possible synergistic impact on residue flexibility and binding, we examined the RMSF profiles of TREM2^WT^ and TREM2^R47H^ (Figure S7B, top and bottom rows, respectively). TREM2^WT^ exhibited multiple flexible regions regardless of ApoE isoform, with regions between CDRs showing the highest flexibility. In contrast, TREM2^R47H^ simulations exhibited distinct RMSF patterns. Three simulations—two with ApoE3, and one with ApoE2—displayed large RMSF shifts across the entire protein, including the CDRs. Two of these simulations (one with ApoE2 and one with ApoE3) involved the opening of the C‐terminal in ApoE, while in the other ApoE3 simulation, TREM2^R47H^ shifted to an alternate binding site on ApoE3. Interestingly, TREM2^R47H^ exhibited markedly reduced RMSF across all protein regions in the presence of ApoE4. This contrasts with TREM2^WT^, where simulations with ApoE4 showed some of the highest RMSF values. The reduction in TREM2^R47H^ flexibility when bound to ApoE4 may further explain the synergistic effects of these two AD‐risk factors, as reduced flexibility in key binding regions in both proteins occurs exclusively in this context.

Following shifts in TREM2^R47H^’s interactions with ApoE, particularly due to the dynamic repositioning of ApoE's C‐terminal, we generated contact maps to identify the interacting residues between TREM2^R47H^ and ApoE (Figure S8). TREM2^R47H^ interactions with ApoE2 involved both the canonical and extended hinge regions, as well as the N‐terminal, closely aligning with results from the TREM2^WT^‐ApoE2 simulations. For ApoE3, interactions occurred at its C‐terminal, again consistent with results from the TREM2^WT^‐ApoE3 simulations. Lastly, with ApoE4, TREM2^R47H^ interacted with the extended hinge region and C‐terminal, contrasting with the TREM2^WT^ simulations where ApoE4 bound via its extended hinge region and N‐terminal. This consistency in ApoE2 and ApoE3 binding sites across TREM2 variants, in contrast to the altered binding of ApoE4, underscores the unique sensitivity of ApoE4 to the *R47H* mutation.

Overall, these structural differences in the TREM2^R47H^‐ApoE4 complex likely stem from the reduced flexibility of TREM2^R47H^’s CDR2 region, caused by α‐helical stabilization, which limits its adaptability in forming diverse binding interfaces. ApoE4 further amplifies this rigidity through its structurally rigid hinge region, which forms a binding pocket that locks TREM2^R47H^ into less adaptable states, suppressing the ability of the TREM2^R47H^‐ApoE4 complex to dynamically reconfigure. This mutual rigidity may underlie the lack of structural changes observed in ApoE4 simulations and contribute to the experimentally observed, synergistic effects of TREM2^R47H^ and ApoE4 on AD pathology.^25^

Finally, synthesizing these findings and directly comparing TREM2^WT^ and TREM2^R47H^, we analyzed binding energies across clusters for each ApoE isoform (Figure S6C for ApoE2; Figure S6D for ApoE3; Figure S6E for ApoE4). First, ApoE2 showed the greatest resistance to decreases in binding affinity due to the *R47H* mutation across all TREM2 clusters. As ApoE2 is known to be protective in AD^70^, ^71^ and often does not exhibit the strongest affinities in our models, this suggests that weak binding, to an extent, is not inherently detrimental. Instead, our simulations indicate that this relatively weak affinity may enable ApoE2 to reconfigure itself and its interactions with TREM2, while still maintaining sufficient binding. Second, Cluster C of TREM2 emerged as the most resistant to decreases in affinity caused by the *R47H* mutation across all ApoE isoforms. Notably, Cluster C most closely resembles the CDR2 conformation in the TREM2^WT^ crystal structure. In contrast, the remaining clusters (A and B) exhibited significant decreases in affinity due to the *R47H* mutation across ApoE isoforms, consistent with prior experiments^34^ showing markedly reduced binding to TREM2^R47H^ compared to TREM2^WT^. Third, the strongest interaction energies of all simulations occurred between WT‐B and ApoE4. Cluster B most closely resembles the CDR2 conformation in the TREM2^R47H^ crystal structure, potentially providing further insights into the synergistic effects of the ApoE4 and TREM2^R47H^ risk factors on AD pathology.

### 3.5 Simulations recapitulate biophysical experiments on TREM2‐ApoE binding

**FIGURE 5:**
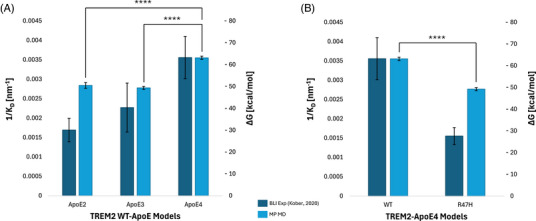
Molecular dynamics simulations recapitulate experimental binding trends of increased TREM2^WT^‐ApoE4 binding and decreased TREM2^R47H^ binding. (A) MD interaction free energies and steady‐state binding affinities^34^ were compared for the TREM2^WT^‐ApoE models. Binding affinities, represented as 1/*K*_D_, were plotted in navy (left‐hand y‐axis). Binding standard deviations are shown with error bars.^32^ The mean free energies for the TREM2^WT^‐ApoE model that represented the most populous (MP) cluster (TREM2^WT^ cluster B for ApoE2 and ApoE4, cluster C for ApoE3) were plotted in light blue, with the right‐hand y‐axis. Energy standard errors of the mean are shown with error bars. Significant differences between free energies are shown (**** *p* < 0.0001). (B) MD interaction free energies and steady‐state binding affinities^34^ were compared for the TREM2‐ApoE4 models. Binding affinities, represented as 1/K_D_, were plotted in navy (left‐hand y‐axis). Binding standard deviations are shown with error bars.^34^ The mean free energies for the TREM2‐ApoE4 model that represented the MP cluster (cluster B for both TREM2^WT^ and TREM2^R47H^) were plotted in light blue, with the right‐hand y‐axis. Energy standard errors of the mean are shown with error bars. Significant differences between free energies are shown (**** *p* < 0.0001).

To rigorously validate the findings from our MD simulations, we compared the results of our MM/PBSA binding free energy calculations to Kober et al.’s biophysical data on unlipidated TREM2‐ApoE interactions.^34^ MM/PBSA is a computational method used to calculate interaction energies between proteins and ligands, producing values highly dependent on simulation‐specific parameters such as the force field and solvation methodology employed.^63^, ^64^ Consequently, these values are used to illustrate relative trends in TREM2‐ApoE interactions across computational and experimental data, rather than being directly correlated with absolute experimental binding constants.^76^ Figure 5A illustrates a comparison between the binding free energies of the three ApoE isoforms for TREM2^WT^ (same values as shown in Figure 3D) and the inverse of the steady‐state affinity (*K*_D_) values measured for human TREM2^WT^ (hTREM2) bound to human ApoE. The experimental K_D_ values were obtained using biolayer interferometry (BLI), where streptavidin biosensors immobilized with hTREM2 were dipped into wells containing purified, recombinant ApoE prior to affinity measurements.^34^ BLI measurements likely reflect the most stable or dominant binding interactions, providing a static snapshot of binding that aligns with the most biologically relevant conformations captured in the simulations.

Thus, for computational comparisons, we used the binding energy values from Figure 3D corresponding to the most populated (MP) cluster (based on CDR2 distance) of each TREM2^WT^‐ApoE combination, reasoning that these clusters likely represent the dominant conformations in experimental measurements due to their highest occurrence in the simulations. Figure S9 presents a similar comparison using average binding free energies for each TREM2^WT^‐ApoE combination. These averages were obtained by weighting each cluster's binding free energy by the number of simulation frames in which the corresponding CDR2 conformation was observed, as indicated in Figure 3D, and summing the three cluster values for each system. The use of average binding free energies provides an ensemble‐based metric, capturing the dynamic contributions of all sampled conformations, including less populated but potentially relevant states. This metric complements experimental BLI data by offering a broader perspective on the binding process that incorporates the full range of configurations sampled during the simulations.

The results demonstrate strong agreement between experiments and simulations, based on the MP conformations, particularly in capturing relative binding trends across the isoforms. For the experimental data, the binding affinities increase in the order ApoE2 < ApoE3 < ApoE4. The simulation data also captures the overall trends, predicting ApoE4 as the strongest binder and ApoE2 and ApoE3 as weaker binders. Additionally, the simulations capture the fold increase in affinity of ApoE4 relative to ApoE2 and ApoE3. Notably, the differences in binding free energy between the MP conformations of ApoE2 and ApoE3 in the simulations are not significant. However, the differences between ApoE2 and ApoE4, as well as between ApoE3 and ApoE4, are significant. This distinction aligns with the experimental data, where relatively large error bars were reported for ApoE2 and ApoE3, but much smaller error bars were observed for ApoE4. These experimental uncertainties support the notion that significant binding affinity differences between ApoE2 and ApoE3 are difficult to resolve experimentally or are perhaps less biologically meaningful, consistent with the simulations.

Importantly, as reported earlier, our simulations revealed that the relatively weak binding affinity of ApoE2 facilitates dynamic reconfiguration of TREM2‐ApoE2 complexes, unlike the more stable interactions observed with ApoE4. This observation, based on ensemble‐level data, reflects differences that may not be captured by the static nature of BLI measurements. As shown in the average binding free energy graph (Figure S9A), ApoE2 binds the most weakly from an ensemble perspective, further emphasizing its dynamic nature compared to ApoE4.

Our simulations also capture the significant decrease in binding affinity of ApoE4 for TREM2^R47H^ compared to TREM2^WT^, consistent with observations from the experiments (Figure 5B; see Figure S9B for weighted averages). Notably, ApoE4 was the only isoform for which experimental binding affinity values to TREM2^R47H^ were explicitly reported. The ability of our simulations to reproduce this reduction highlights their utility in accurately modeling the effects of specific mutations on TREM2‐ApoE interactions.

## 4 DISCUSSION

TREM2 and ApoE interactions are implicated in AD pathology but remain poorly understood at the molecular level, necessitating an atomistic, physics‐based approach like MD simulations. Prior MD studies focused on relatively short timescales and selective ApoE isoforms, leaving significant gaps in our understanding.^35^, ^36^ Herein, we employed long‐timescale MD to probe the structural dynamics of TREM2 and all ApoE isoforms, revealing critical insights into their interactions.

Our findings highlight the importance of TREM2's CDR2 dynamics in ligand binding, which may determine pathogenicity. We observed that TREM2^WT^ and TREM2^R47H^ can both access “open” CDR2 states, although with differing stability and frequency. TREM2^WT^’s CDR2 exhibited substantial fluctuations in Loop 1 and 2 distances (metrics we defined to characterize CDR2 movement), potentially facilitating diverse ligand binding. Comparatively, TREM2^R47H^ showed restricted—but not fully eliminated—CDR2 dynamics, with a reduced frequency of sampling wide‐ranging states due to sustained α‐helicity, possibly limiting its ligand‐binding capabilities, consistent with observations in computational, cellular, and animal models with such risk‐associated mutations.^14^, ^17^, ^18^, ^19^

Our findings challenge the notion that an open CDR2 conformation is solely pathogenic,^14^, ^19^, ^68^, ^69^ suggesting instead that suppressed CDR2 dynamics in TREM2^R47H^ underlie impaired ligand binding and pathogenic TREM2 behavior. The apparent stochasticity in sampling open CDR2 states highlights the need for additional long‐timescale simulations to determine accurate frequencies of accessing these states across TREM2^WT^ and TREM2^R47H^, which likely plays a crucial role in ligand binding, TREM2 function, and ultimately disease progression.

Our simulations also revealed distinct structural differences among ApoE isoforms, particularly in ApoE4, which maintained a longer C‐terminal α‐helix and higher β‐sheet content in its hinge region. These features may contribute to ApoE4's higher aggregation propensity and stronger TREM2 affinity.^6^, ^31^, ^34^, ^76^ The longer α‐helix may promote oligomerization, as these residues form the known C‐terminal interface enabling ApoE oligomerization,^32^ while increased β‐sheet content could stabilize the hinge and enhance TREM2‐ApoE4 affinity, given recent experimental evidence that TREM2 binds ApoE's hinge.^34^ ApoE4's hinge also exhibited greater self‐interaction and a more centralized, globular binding interface compared to ApoE2 and ApoE3, which may also facilitate TREM2 binding. Further investigation is needed to clarify these behaviors and their effects on ApoE lipidation, as ApoE4 is poorly lipidated relative to other isoforms, an effect exacerbated as AD pathology progresses.^6^, ^77^, ^78^ Disruption of secondary structures—particularly within lipoprotein receptor binding sites—or the formation of unexpected structures like β‐sheets may play a larger role in ApoE's function than previously realized.

The third helix in ApoE's extended hinge, consistently maintained across all isoforms, appears crucial for mediating TREM2 binding, aligning with biophysical evidence that residues 192–238 in this region have higher TREM2 affinity.^34^ In contrast, the canonical hinge frequently lost secondary structure in the simulations, suggesting a lesser role in ApoE‐TREM2 interactions. This transition from a structured α‐helix to a flexible, disordered region—and its potential contribution to nearby intrinsically disordered regions—highlights these regions’ importance for ApoE's conformational dynamics and lipid‐binding properties.^29^ Loss of α‐helicity in the C‐terminal may also affect lipid binding, as ApoE's lipid‐binding site (V244‐A277) resides within this region. Whether this α‐helix is directly required for lipid binding or primarily facilitates conformational changes that optimize binding interfaces remains unclear.

Expanding on these findings, our analysis of TREM2^WT^‐ApoE complexes revealed that the affinity of different ApoE isoforms influences complex configuration, with distinct functional implications. ApoE2, associated with a protective role in AD,^70^, ^71^ exhibited weaker affinity for TREM2^WT^, consistent with experiments.^34^ Visual trajectory analysis suggested this weaker affinity may permit more dynamic reconfigurations, enabling TREM2^WT^ to adopt binding conformations with ApoE2 that differ substantially from those observed with ApoE4, a known stronger binder. Such flexibility could be essential for effective microglial signaling. Contrastingly, ApoE4 demonstrated limited capacity to modulate the TREM2^WT^‐ApoE4 complex within the simulated timescales, potentially restricting dynamic reconfigurations.

When investigating the impact of the TREM2^R47H^ variant on ApoE interactions, we found that TREM2^R47H^ binds less effectively to all ApoE isoforms compared to TREM2^WT^, consistent with experiments.^10^, ^11^, ^72^ The *R47H* mutation altered CDR2 dynamics, causing fluctuations in Loop 1 and 2 distances and unstable ApoE binding. For example, in one simulation, ApoE2 dissociated entirely from TREM2^R47H^ before dynamically reconfiguring and attempting to rebind in a new orientation—a behavior not observed with TREM2^WT^, highlighting the mutation's impact on CDR2 flexibility. Moreover, simulations of TREM2^R47H^ bound to ApoE3 showed that, although ApoE3 interacted with multiple TREM2 regions (including CDR2), these interactions were less stable overall. This underscores how *R47H* compromises TREM2's ability to maintain stable and effective ligand interactions. Additionally, TREM2^R47H^, already characterized by reduced CDR2 flexibility due to α‐helical stabilization, becomes further restricted upon ApoE4 binding. ApoE4's structurally rigid hinge regions rigidify the complex, locking TREM2^R47H^ into less adaptable conformations that suppress its ability to dynamically reconfigure.

Collectively, our findings suggest that, while ApoE4 may not always induce pathogenic effects when interacting with TREM2^WT^, it tends to remain in conformational states that the more structurally dynamic isoforms, particularly ApoE2, do not. The isoform‐specific nature of these interactions aligns with ApoE4's known pathogenic effects in AD, as its rigidifying influence contrasts with ApoE2's promotion of flexibility and adaptability in TREM2 complexes. Overall, our results indicate that the mutual reduction in flexibility within the TREM2^R47H^‐ApoE4 complex underlies their synergistically detrimental effects in AD.^25^ This combined suppression of CDR2 flexibility may amplify pro‐inflammatory signaling, providing a mechanistic basis for their cooperative contributions to AD pathology.

Importantly, there are several limitations in our study, partly due to its computational nature. Like previous MD studies,^14^, ^67^, ^68^, ^69^ our work assumes TREM2's Ig‐like domain is representative of membrane‐bound TREM2, which also contains the flexible stalk, transmembrane helix, and a cytosolic tail. However, since we found that TREM2‐ApoE interactions primarily involve their CDRs and hinge regions—which remain accessible when TREM2 is bound to co‐adaptors and the membrane—our analyses reflect physiologically relevant protein‐protein binding.

Another potential limitation concerns TREM2 glycosylation. TREM2's ectodomain is glycosylated at two sites (N20, N79). Our study utilized a human TREM2^WT^ structure glycosylated at these sites during crystallization^18^ (though precise glycan chemistries were not reported) and a human TREM2^R47H^ structure expressed in *Escherichia coli*, lacking glycosylation altogether.^19^ Despite these expression differences, we posited that using the experimentally‐derived TREM2^R47H^ structure offered greater biological relevance for our simulations than introducing *R47H* directly into TREM2^WT^’s structure. Supporting this approach, Sudom et al. analyzed NAG glycan interactions with TREM2^WT^ and TREM2^R47H^ and concluded that structural differences were not due to expression or glycosylation,^19^ noting significant variation in NAG orientations on TREM2^WT^. Similarly, another study identified differing glycosylation profiles between TREM2^WT^ and TREM2^R47H^ ^80^, though the exact glycan compositions remain unknown. Given this diversity and lingering uncertainty surrounding TREM2's glycans, we excluded them from our simulations to avoid introducing non‐physiological structural changes. Moreover, while N79 is near CDR2, it lies at the opposite end of the hydrophobic tip encompassing CDR1, CDR2, and CDR3, making it unlikely to induce significant conformational changes in this region. Nonetheless, future studies should incorporate more realistic models of full‐length, fully‐glycosylated TREM2 embedded in a lipid membrane with its co‐adaptors.

We performed all simulations in triplicate, from 500 to 1000 ns—longer than any previous simulations published to date from other groups^35^, ^36^—and are thus confident the conformations accessed, albeit stochastic, are functionally relevant. However, TREM2's apparent stochastic behavior should be tested with more replicates to fully characterize CDR2 dynamics. Using a single representative structure for each ApoE isoform is another limitation, but given the high computational demands of long‐timescale simulations, we prioritized generating representative ApoE structures to explore a broader range of TREM2 conformations. Furthermore, rigid docking, as used herein, does not fully capture protein‐protein interaction dynamics. Nevertheless, our long‐timescale simulations allowed ample time for proteins to reconfigure initial binding poses and achieve more favorable conformations. Biases may arise from selecting docked models, although we addressed this by running triplicate simulations with different TREM2 structures bound to various ApoE‐binding sites. Future work should explore more dynamic docking models, with simulations spanning a broader range of starting positions and configurations for both TREM2 and ApoE.

Our study also utilized monomeric, unlipidated ApoE, derived from a mutated form of ApoE3, with these mutations reverted prior to simulation and additional mutations inserted to distinguish isoforms. ApoE isoform dynamics would likely be more accurate with experimentally resolved structures for each isoform. Moreover, while unlipidated ApoE is known to exist in the brain, it is expected to be more commonly observed in its tetrameric rather than monomeric form. Although no experimental structures of tetrameric ApoE currently exist, future in silico studies could aim to model this form to better recapitulate experimental conditions for studying ApoE‐TREM2 interactions.

Finally, Kober et al.^34^ and Greven et al.^80^ studied several TREM2 variants in the context of ApoE4 binding. While we focused on the AD‐risk variant TREM2^R47H^, recent BLI experiments showed that other TREM2 mutations within the hydrophobic patch alter or completely inhibit ApoE binding.^34^, ^80^ Specifically, mutations in CDR1 (M41, W44) caused slight decreases in ApoE4 binding, illustrating the importance of hydrophobic residues at the CDR1 interface known to interact with ApoE. More strikingly, mutations to hydrophobic residues in CDR2 (L69, W70, F74)—all within CDR2's α‐helical region—significantly inhibited binding, further emphasizing the critical role of CDR2 α‐helicity. These findings suggest that maintaining or losing secondary structure in this region can substantially affect TREM2‐ApoE interactions. Future in silico studies should explicitly simulate these TREM2 mutations to determine how the resulting conformational changes influence ApoE binding, further expanding our understanding of the impact of CDR2 flexibility and α‐helicity on binding behavior.

In conclusion, the novel insights gained from our simulations underscore the complexity of TREM2‐ApoE interactions and highlight the need for further studies to explore additional risk‐associated TREM2 mutations and the impact of ApoE lipidation on its structure, function, and ability to bind TREM2. It is also crucial to study TREM2 binding to alternate ligands that promote distinct signaling cascades essential to microglial health and function.^15^, ^18^, ^19^, ^21^, ^22^ Such studies are critical for advancing our understanding of protein‐protein interactions in neurodegenerative disease settings and could pave the way for new therapeutic strategies.

## CONFLICT OF INTEREST STATEMENT

The authors have no conflicts of interest to declare. Author disclosures are available in the supporting information.

## Supporting information



Supporting Information

ICMJE Disclosure Form

## Data Availability

The molecular dynamics coordinates, trajectories, and structures utilized in this study are available upon reasonable request to the corresponding authors. All other pertinent data for this research are provided within the article and its supplementary materials.
