# Supplementary material for: Synergistic reduction in interfacial flexibility of TREM2R47H and ApoE4 may underlie AD pathology
Source: Alzheimers Dement. 2025 Apr 12;21(4):e70120. doi: 10.1002/alz.70120 (PMC11992651; doi:10.1002/alz.70120)
Supplement: Supplementary file 1 — Supporting Information [file ALZ-21-e70120-s002.pdf]

SUPPLEMENTAL INFORMATION

Synergistic reduction in interfacial flexibility of TREM2^{R47H} and ApoE4 may underlie AD pathology

Emma E. Lietzke[1,2], David Saeb[1], Emma C. Aldrich[1], Kimberley D. Bruce[2], and Kayla G. Sprenger[1]**

[1]Department of Chemical and Biological Engineering, University of Colorado Boulder, Boulder, CO, USA
[2]Department of Endocrinology, Metabolism, and Diabetes, University of Colorado Anschutz Medical Campus, Aurora, CO, USA

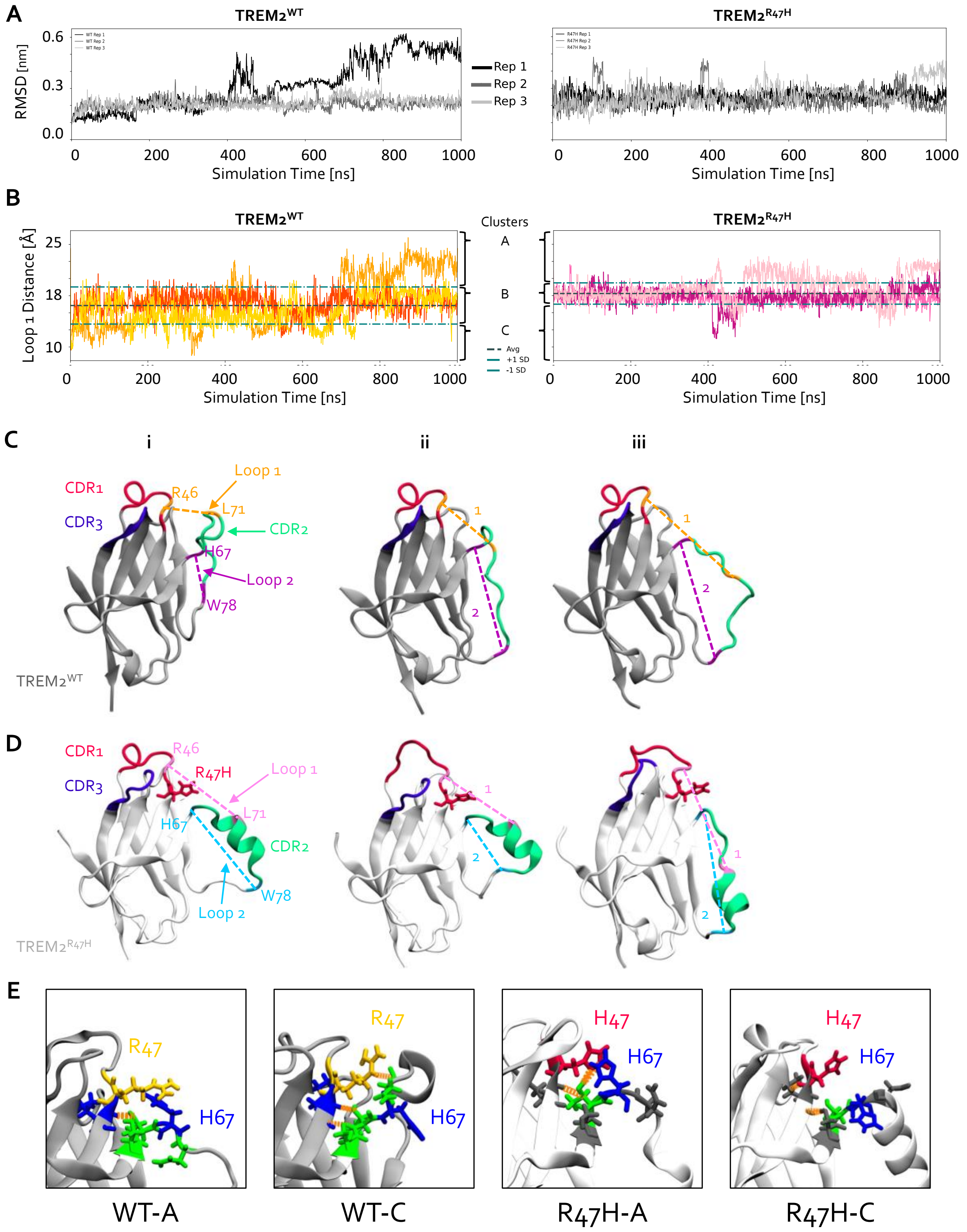

Figure S1. TREM2 conformation clustering. (A) RMSD of TREM2^{WT} (left) and TREM2^{R47H} (right) simulations vs. time. (B) Loop 1 distance plots of TREM2^{WT} (left) and TREM2^{R47H} (right) vs time. The average distance and its standard deviation are shown. Clusters A, B, and C are shown, separated by the standard deviations. For TREM2^{WT}, the cut-off bounds (in Å) were: A > 18.7725; 18.7725 ≥ B ≥ 13.3499; C < 13.3499. For TREM2^{R47H}, the cut-off bounds (in Å) were: A > 19.3606; 19.3606 ≥ B ≥ 16.2480; C < 16.2480. (C-D) Snapshots of TREM2^{WT} (C) and TREM2^{R47H} (D) from three simulation timepoints, as indicated in (Fig. 1B) and (Fig. 1C): i (0 ns), ii (625 ns), and iii (1000 ns). CDR, CDR2, and CDR3 are colored as in red, green, and blue, respectively. Loops 1 and 2 are shown for TREM2^{WT} in orange and purple; Loops 1 and 2 are shown for TREM2^{R47H} in pink and cyan, respectively. (E) Snapshots of

TREM2 residue interactions between CDR1 and CDR2. $TREM2^{WT}$ is represented in light gray, with residues of interest represented in licorice. R47 is in yellow, basic sidechains (K48, H67) in blue, and polar sidechains (S65, T66, N68) in green. Hydrogen bonds (hydrogens within a cutoff distance of 4 Å) are shown in the orange dotted lines. $TREM2^{R47H}$ is shown in white. H47 is in red, and experimentally predicted H-bonding sidechains (T66, H67) are shown in green and blue, resp. The residues predicted to only interact in H-bonding in $TREM2^{WT}$ are shown in dark gray (K48, S65, N68).

A Null hypothesis: $\mu_{ApoE2} = \mu_{ApoE3} = \mu_{ApoE4}$, where μ = the true mean of the number of residues in Full Hinge across [900,1000] ns

Full Hinge Residues in β-Sheet		
Pairwise Tukey HSD Test, FWER = 0.05<br>Multiple Comparison of True Means		
Group 1	Group 2	Significant Difference
ApoE2	ApoE3	*
ApoE2	ApoE4	***
ApoE3	ApoE4	***

Full Hinge Residues in RH α-Helix		
Pairwise Tukey HSD Test, FWER = 0.05<br>Multiple Comparison of True Means		
Group 1	Group 2	Significant Difference
ApoE2	ApoE3	--
ApoE2	ApoE4	***
ApoE3	ApoE4	***

Full Hinge Residues in LH α-Helix		
Pairwise Tukey HSD Test, FWER = 0.05<br>Multiple Comparison of True Means		
Group 1	Group 2	Significant Difference
ApoE2	ApoE3	***
ApoE2	ApoE4	--
ApoE3	ApoE4	***

B

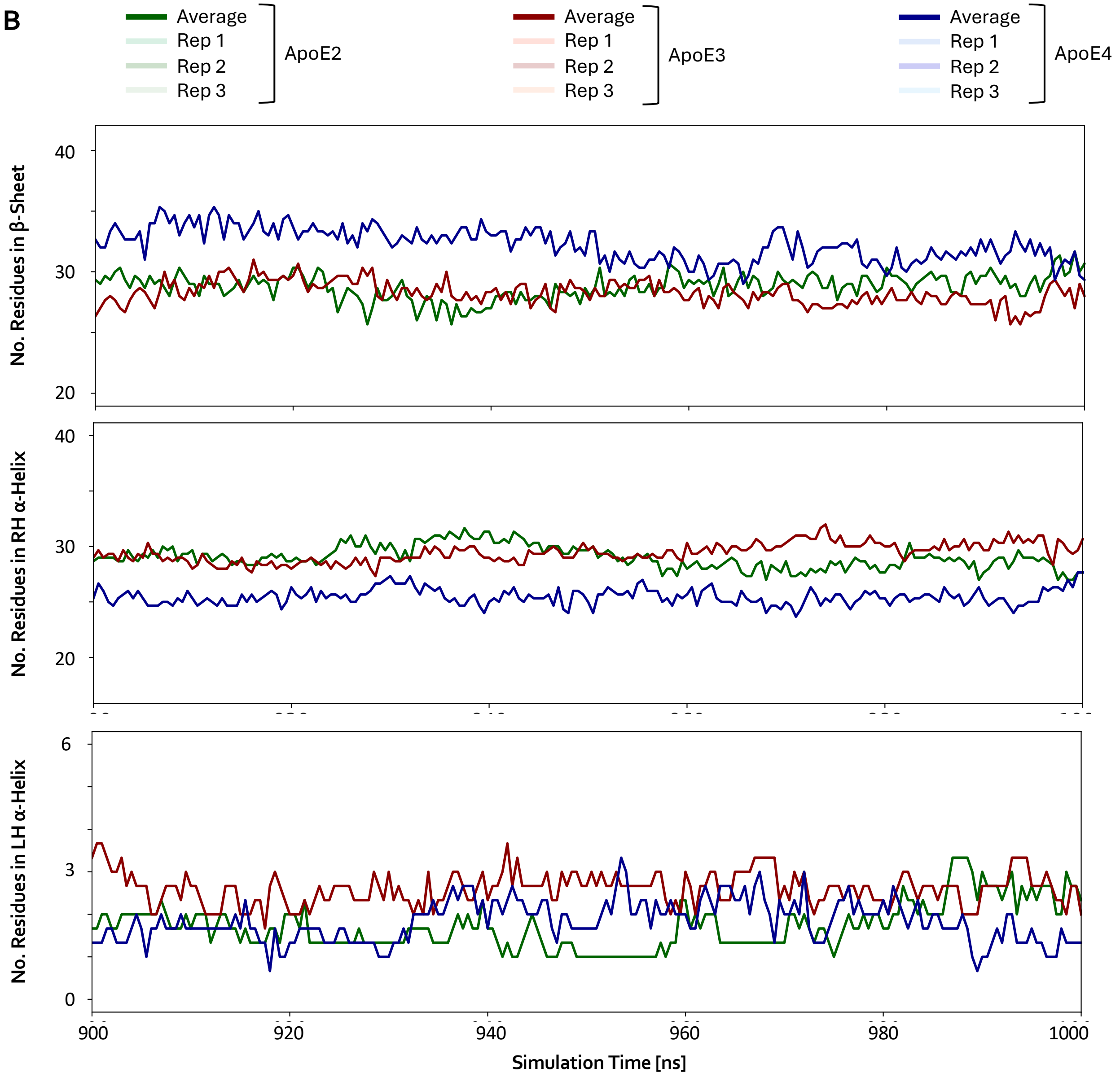

Figure S2. Secondary structure of ApoE hinge varies across isoform. (A) ANOVA post-hoc pairwise Tukey HSD tests were performed on the number of hinge residues involved in each type of secondary structure across ApoE isoforms. The true mean of the hinge residues involved in a structure was calculated across the nine simulations (triplicates for each ApoE isoform). The statistically significant differences are shown for each group comparison (* $p < 0.05$, *** $p < 0.001$). (B) Temporal plots of the number of Cα residues expected to be involved in β-sheets, RH α-

helixes, and LH α-helices for each replicate for hinge residues in ApoE2, ApoE3 and ApoE4 simulations (9 total simulations). Values for the replicates are shown in the light greens (ApoE2), light reds (ApoE3), and light blues (ApoE4). The averaged values for each ApoE over time are shown in the dark green, dark red, and dark blue. To determine the number of residues in each secondary structure, the number of ApoE Cα residues within the ψ and φ angles of the pre-determined bounds for β-sheets, RH α-helices, and LH α-helices. These were counted for each 0.5 ns timepoint between 900 to 1000 ns of the simulations, as these were the converged portions of the RMSD plots (Fig. 2A, C, E). The bounds for the β-sheet were: φ = [-180,-20], ψ = [70,180]. The bounds for the RH α-helix were: φ = [-140,-20], ψ = [-70,40]. The bounds for the LH α-helix were: φ = [20,140], ψ = [-50,85].

A Null hypothesis: $\mu_{ApoE2} = \mu_{ApoE3} = \mu_{ApoE4}$, where μ = the true mean of the number of residues in C-terminal across [900,1000] ns

C-terminal Residues in β-Sheet		
Pairwise Tukey HSD Test, FWER = 0.05<br>Multiple Comparison of True Means		
Group 1	Group 2	Significant Difference
ApoE2	ApoE3	***
ApoE2	ApoE4	***
ApoE3	ApoE4	***

C-terminal Residues in RH α-Helix		
Pairwise Tukey HSD Test, FWER = 0.05<br>Multiple Comparison of True Means		
Group 1	Group 2	Significant Difference
ApoE2	ApoE3	***
ApoE2	ApoE4	*
ApoE3	ApoE4	***

C-terminal Residues in LH α-Helix		
Pairwise Tukey HSD Test, FWER = 0.05<br>Multiple Comparison of True Means		
Group 1	Group 2	Significant Difference
ApoE2	ApoE3	***
ApoE2	ApoE4	***
ApoE3	ApoE4	***

B

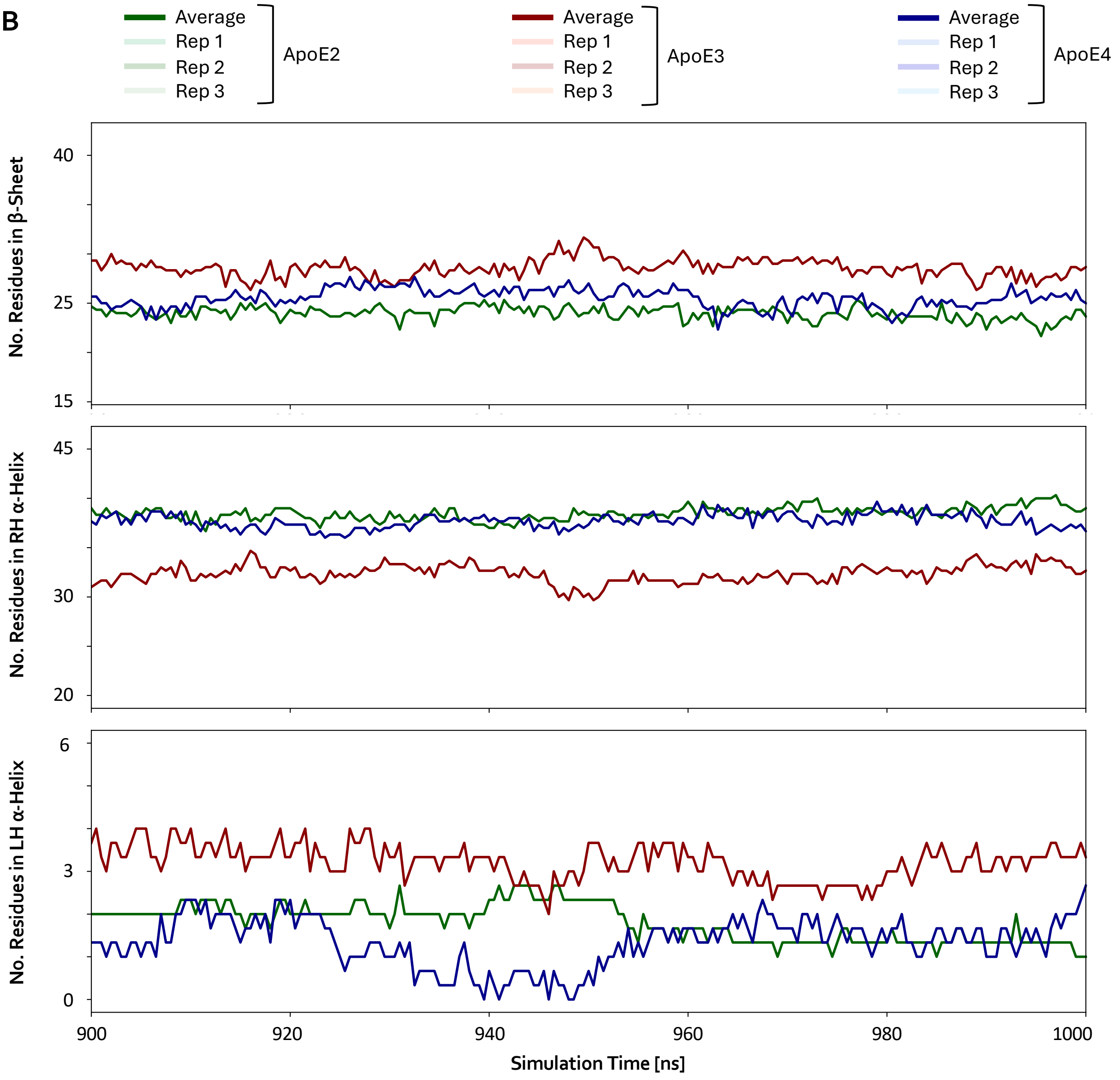

Figure S3. Secondary structure of ApoE C-terminal varies across isoform. (A) ANOVA post-hoc pairwise Tukey HSD tests were performed on the number of C-terminal residues involved in each type of secondary structure across ApoE isoforms. The true mean of the C-terminal residues involved in a structure was calculated across the nine simulations (triplicates for each ApoE isoform). The statistically significant differences are shown for each group comparison (* p < 0.05, *** p < 0.001). (B) Temporal plots of the number of Cα residues expected to be involved in

β-sheets, RH α-helixes, and LH α-helices for each replicate for C-terminal residues in ApoE2, ApoE3 and ApoE4 simulations (9 total simulations). Values for the replicates are shown in the light greens (ApoE2), light reds (ApoE3), and light blues (ApoE4). The averaged values for each ApoE over time are shown in the dark green, dark red, and dark blue. To determine the number of residues in each secondary structure, the number of ApoE Cα residues within the ψ and φ angles of the pre-determined bounds for β-sheets, RH α-helices, and LH α-helices. These were counted for each 0.5 ns timepoint between 900 to 1000 ns of the simulations, as these were the converged portions of the RMSD plots (Fig. 2A, C, E). The bounds for the β-sheet were: φ = [-180,-20], ψ = [70,180]. The bounds for the RH α-helix were: φ = [-140,-20], ψ = [-70,40]. The bounds for the LH α-helix were: φ = [20,140], ψ = [-50,85].

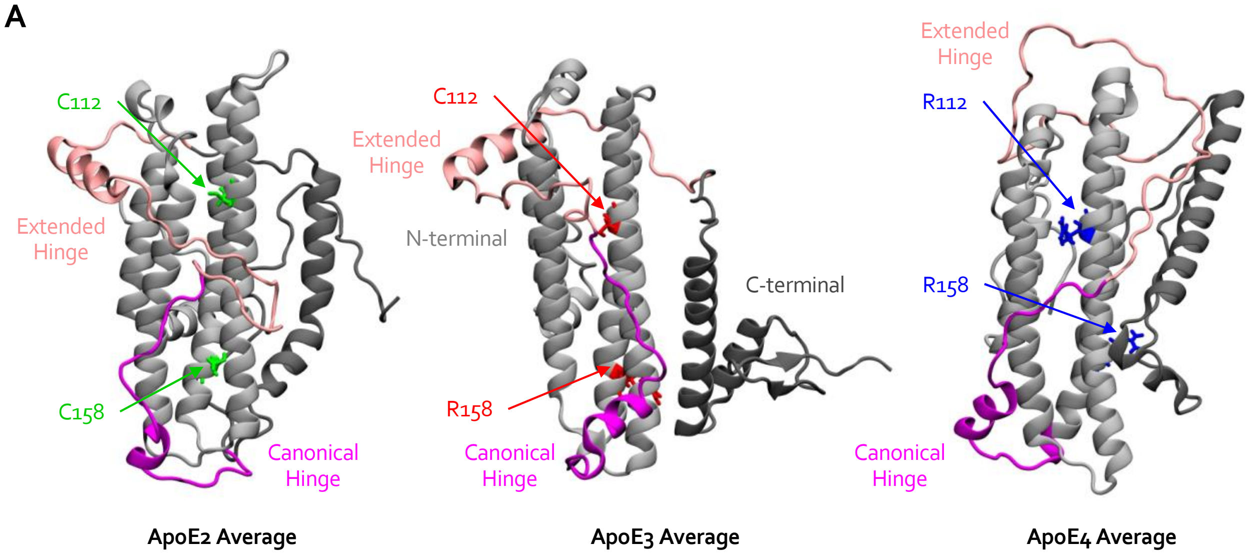

Figure S4. Representative structure for ApoE2, ApoE3, and ApoE4. (A) The average (representative) structures of ApoE2, ApoE3, and ApoE4 are shown in new cartoon representation. The N-terminal is colored in light gray and the C-terminal in dark gray. The canonical hinge is colored in magenta and the extended hinge in light pink. Residues 112 and 158 are colored in green, red, and blue, for ApoE2, ApoE3, and ApoE4, respectively.

Percentage of Total TREM2 CDR Residues within 4 Å of ApoE Full Hinge Residues for the 5 Top-Scoring ClusPro Output Conformations

	Models	WT_A	WT_B	WT_C	R47H_A	R47H_B	R47H_C
ApoE2	0	38.46%	38.46%	42.31%	30.77%	3.85%	15.38%
	1	42.31%	57.69%	34.62%	11.54%	42.31%	42.31%
	2	0%	3.85%	11.54%	30.77%	19.23%	7.69%
	3	30.77%	3.85%	46.15%	23.08%	3.85%	3.85%
	4	38.46%	11.54%	3.85%	11.54%	7.69%	26.92%
ApoE3	0	11.54%	0%	7.69%	0%	0%	0%
	1	0%	7.69%	7.69%	0%	57.69%	0%
	2	0%	0%	7.69%	3.85%	0%	3.85%
	3	0%	0%	0%	0%	0%	0%
	4	3.85%	0%	0%	0%	0%	0%
ApoE4	0	0%	50.00%	19.23%	19.23%	0%	0%
	1	7.69%	11.54%	0%	0%	0%	0%
	2	0%	0%	0%	7.69%	0%	0%
	3	65.38%	3.85%	11.54%	0%	0%	0%
	4	0%	0%	30.77%	0%	15.38%	0%

Figure S5. Clustering outputs with biologically relevant interacting residues between TREM2 and ApoE. The percentage of TREM2 CDR residues that were within an interaction distance of 4 Å of the ApoE hinge were calculated for the top 5-scoring models for each TREM2/ApoE combination. The percentages are shown in the table, and the model picked for simulations is highlighted in green, red, and blue, for ApoE2, ApoE3, and ApoE4-based simulations, respectively.

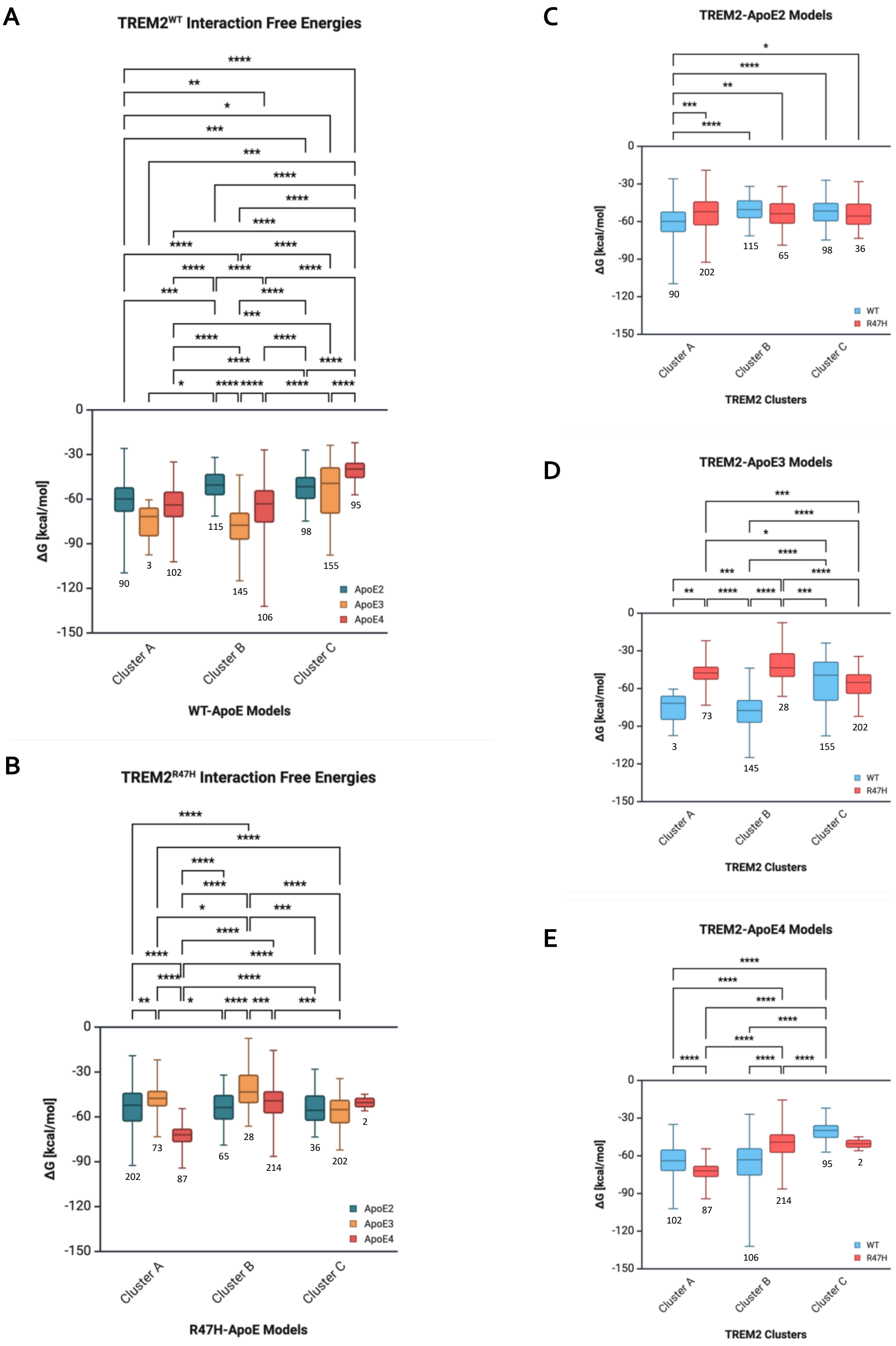

A
TREM2^WT Interaction Free Energies
ΔG [kcal/mol]
0
-30
-60
-90
-120
-150
90
3
102
115
145
106
98
155
95
ApoE2
ApoE3
ApoE4
Cluster A
Cluster B
Cluster C
WT-ApoE Models
B
TREM2^R47H Interaction Free Energies
ΔG [kcal/mol]
202
73
87
65
28
214
36
202
2
ApoE2
ApoE3
ApoE4
Cluster A
Cluster B
Cluster C
R47H-ApoE Models
C
TREM2-ApoE2 Models
ΔG [kcal/mol]
90
202
115
65
98
36
WT
R47H
Cluster A
Cluster B
Cluster C
TREM2 Clusters
D
TREM2-ApoE3 Models
ΔG [kcal/mol]
3
73
145
28
155
202
WT
R47H
Cluster A
Cluster B
Cluster C
TREM2 Clusters
E
TREM2-ApoE4 Models
ΔG [kcal/mol]
102
87
106
214
95
2
WT
R47H
Cluster A
Cluster B
Cluster C
TREM2 Clusters

Figure S6. Interaction free energy calculations for TREM2-ApoE models. Interaction energies were calculated for each 0.5 ns frame, from 450-500 ns, for each of the 18 simulations (n=1,818). Energies are shown in box plots, with the true mean, standard deviation, and range of values. The number of samples (frames) for each group are shown for each, respectively. Two-way ANOVA with Tukey HSD multiple comparison tests were performed for all groups, with all significant differences shown (* $p < 0.05$, ** $p < 0.01$, *** $p < 0.001$, **** $p < 0.0001$). (A) Interaction free energies for $TREM2^{WT}$-ApoE simulations (n=909). Energies are shown for each $TREM2^{WT}$ conformation (Clusters A/B/C) per each ApoE isoform (ApoE2/3/4), from the nine simulations. (B) Interaction free energies for $TREM2^{R47H}$-ApoE simulations (n=909). Energies are shown for each $TREM2^{R47H}$ conformation (Clusters A/B/C) per each ApoE isoform (ApoE2/3/4), from the nine simulations. (C) Interaction free energies for TREM2-ApoE2 simulations (n=606). Energies are shown for each TREM2 conformation (Clusters A/B/C) per TREM2 variant (WT, R47H), from the six simulations. (D) Interaction free energies for TREM2-ApoE3 simulations (n=606). Energies are shown for each TREM2 conformation (Clusters A/B/C) per TREM2 variant (WT, R47H), from the six simulations. (E) Interaction free energies for TREM2-ApoE4 simulations (n=606). Energies are shown for each TREM2 conformation (Clusters A/B/C) per TREM2 variant (WT, R47H), from the six simulations.

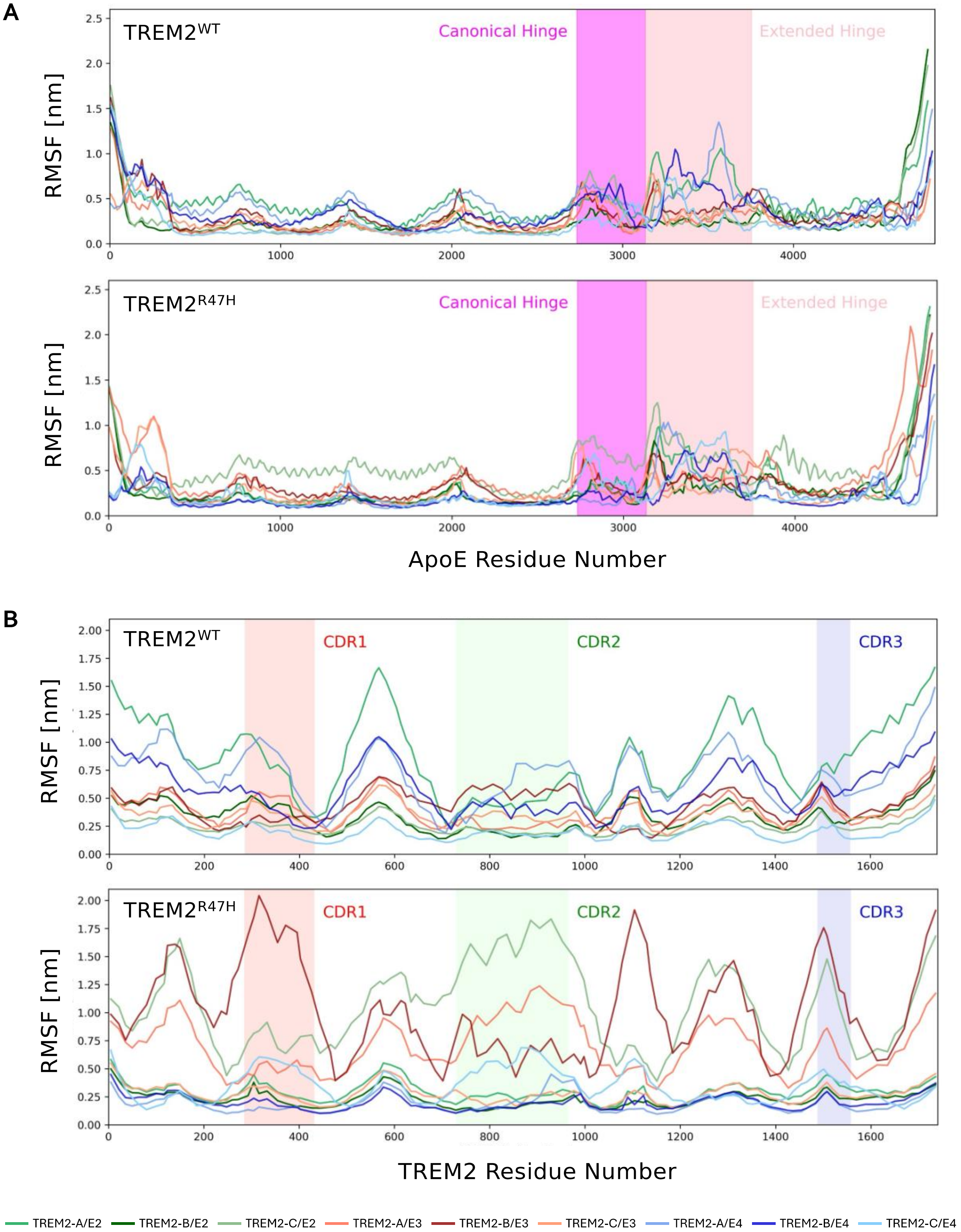

Figure S7. RMSF calculations for TREM2 variants and ApoE isoforms over the entire simulation period. (A) RMSF of ApoE, on the y-axis, was measured for each ApoE residue (x-axis) over the entire simulation period. The canonical and extended hinge regions for ApoE are shown in magenta and light pink, respectively. Simulations with ApoE2 are shown in green, ApoE3 in red, and ApoE4 in blue. In the top graph, the RSMF of each ApoE is plotted for the simulations with TREM2^{WT}. In the bottom graph, the RMSF of each ApoE is plotted for the simulations with TREM2^{R47H}. (B) RMSF of TREM2, on the y-axis, was measured for each TREM2 residue (x-axis) over the entire simulation period. CDR1, CDR2, and CDR3 for TREM2 are shown in red, green, and blue, respectively. Simulations

with ApoE2 are shown in green, ApoE3 in red, and ApoE4 in blue. In the top graph, the RSMF values represent simulations of $TREM2^{WT}$. In the bottom graph, the RMSF values represent simulations of $TREM2^{R47H}$.

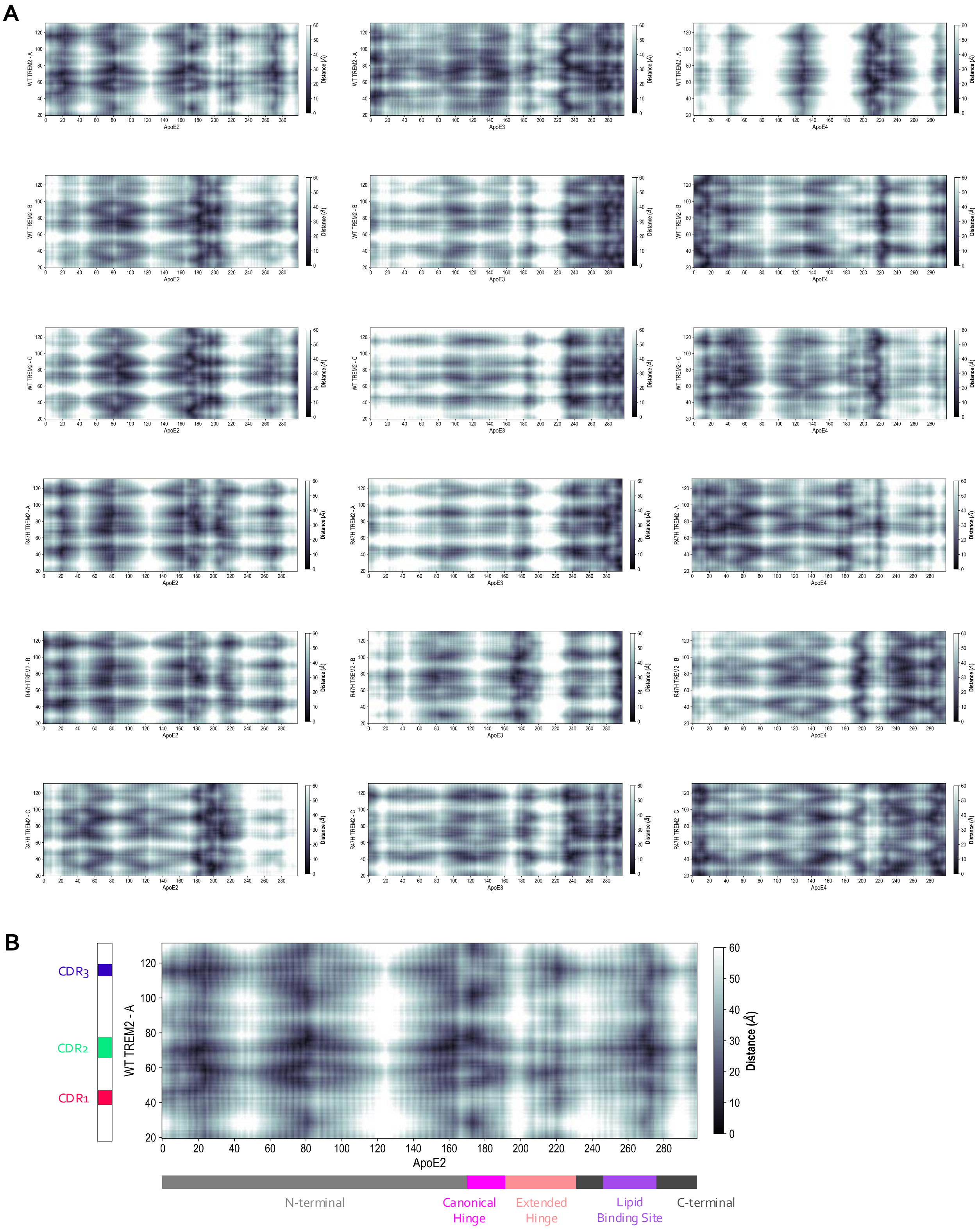

Figure S8. Contact maps of residue interactions between TREM2 and ApoE for each simulation. (A) Distance between each residue of TREM2 and each residue of ApoE was determined for the last 50 ns of each simulation. TREM2 residues are plotted on the y-axis and ApoE on the x-axis. Distances are colored on a scale, as seen on the bar of the right of each plot. (B) Reference contact map is shown, with regions of TREM2 and ApoE colored on the axes. For TREM2, CDR1 is shown in red, CDR2 in green, CDR3 in blue, and the remaining regions in white. For ApoE, the N-terminal is shown in light gray, the canonical hinge in magenta, the extended hinge in light pink, the C-terminal

in dark gray, and the lipid binding site in purple.

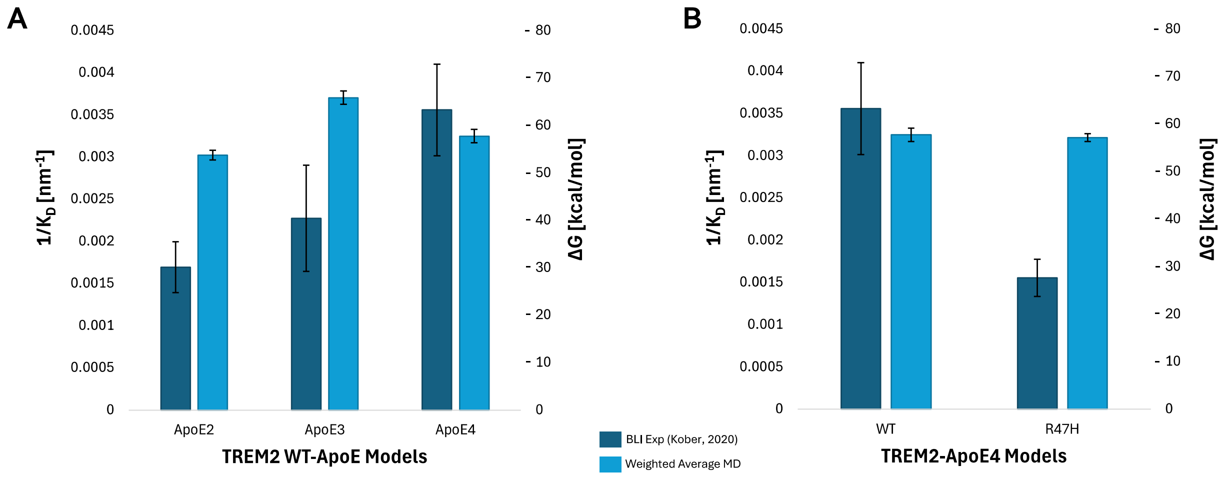

Figure S9. MD interaction free energies and steady-state binding affinities[34] were compared for the TREM2^{WT}-ApoE models. (A) Binding affinities, represented as $1/K_D$, were plotted in navy (lefthand y-axis). Binding standard deviations are shown with error bars[34]. For MD simulations, the weighted interaction energy value was calculated by including all free energies for each TREM2^{WT} cluster with the number of frames that cluster represented. The weighted free energies for the TREM2^{WT}-ApoE model were plotted in light blue, with the righthand y-axis. Energy standard errors of the mean are shown with error bars. (B) Binding affinities, represented as $1/K_D$, were plotted in navy (lefthand y-axis). Binding standard deviations are shown with error bars[34]. For MD simulations, the weighted interaction energy value was calculated by including all free energies for each TREM2^{WT} and TREM2^{R47H} cluster, respectively, with the number of frames that cluster represented. The weighted free energies for the TREM2-ApoE4 model were plotted in light blue, with the righthand y-axis. Energy standard errors of the mean are shown with error bars.
